# Machine Learning–Enhanced LC–HRMS Workflows for Suspect and Nontargeted Screening of Pesticide Residues in Fruits and Vegetables

**DOI:** 10.1021/acs.analchem.5c06726

**Published:** 2026-08-24

**Authors:** Sergio González, Gabriel Merino, Jairo Arturo Guerrero Dallos

**Affiliations:** † Departamento de Química, 28021 Universidad Nacional de Colombia, 111321 Bogotá D.C., Colombia; ‡ Departamento de Física Aplicada, 130299 Centro de Investigación y de Estudios Avanzados, 97310 Mérida, México

## Abstract

Analytical methodologies are essential for detecting and quantifying contaminants. While methods for known compounds are well established, identifying unexpected or unknown compounds remains challenging due to the lack of reference data. Several strategies have been proposed to integrate analytical information into data analysis workflows, but their implementation often requires programming skills and results are rarely presented in a format familiar to analytical chemists, such as the uncertainty budgets used in quantitative analysis. We developed an integrated workflow for suspect screening and nontargeted identification of pesticides in fruits and vegetables using QuEChERS extraction and HPLC-ESI-HRMS (Orbitrap) analysis. The workflow was developed using variable data-independent acquisition (vDIA) data and evaluates protonated and deprotonated species for compound identification. The workflow estimates missing identification properties using machine learning and combines their contributions into a single confidence value. Validation with fortified matrix extracts at different mass concentrations and with real samples containing pesticide residues showed performance comparable to existing software, particularly improving nontargeted identification at high concentrations (>6% increase in identified compounds). This approach reduces the input required for suspect screening and estimates properties for candidate compounds in nontargeted analysis. Results are reported with explicit consideration of the influence of identification properties, analogous to uncertainty budgets in chemical metrology. This workflow improves the interpretability and reliability of chemical identification and supports data-driven decision-making in routine analysis.

## Graphical abstract

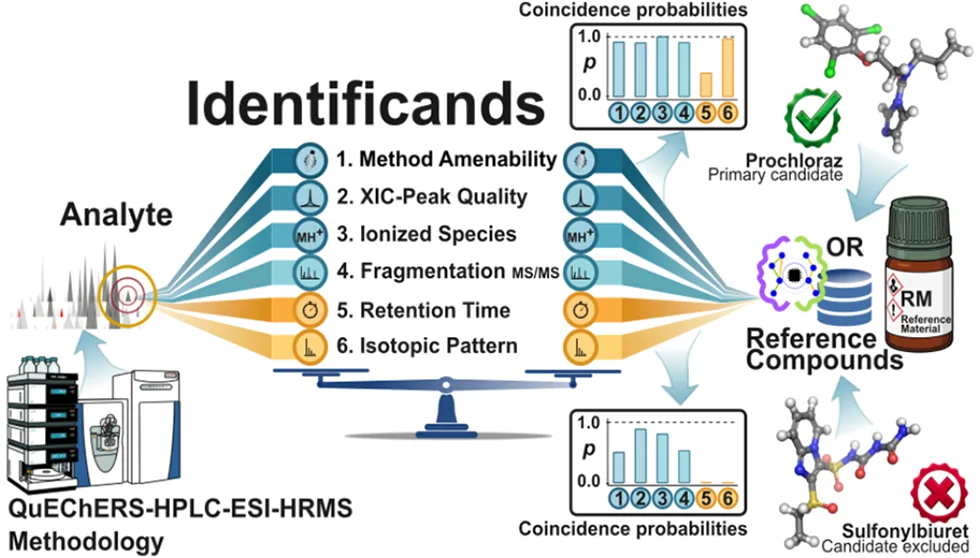

The increasing presence of chemical contaminants in the environment is a major global concern,^1^ driven by population growth and the sustained demand for new products and services. To address this issue, authorities and organizations at different levels have established monitoring programs to detect contaminants and support decisions regarding substances that may affect human health and the environment.^2,3^ The analysis of these substances in environmental samples and food products remains challenging because of the matrix complexity. Nevertheless, advances in instrumental techniques have improved detection capabilities. Hyphenated techniques, such as liquid or gas chromatography coupled with high-resolution mass spectrometry (HRMS), allow the characterization of a broad range of chemical compounds. In the food sector, the surveillance of pesticide residues is particularly important for ensuring food quality and safety, regulatory compliance, and environmental protection. High-performance liquid chromatography (HPLC) coupled with electrospray ionization (ESI) and HRMS-Orbitrap analyzer^4^ has become a versatile platform for the multiresidue analysis of pesticides and other compounds, including veterinary drugs, cosmetic ingredients, and pharmaceuticals.

The adoption of wide-scope methods has expanded the ability of analytical laboratories to detect both known and unknown compounds. Known compounds can be reliably confirmed using targeted analysis (TA) approaches, which rely on target compound lists and reference materials (RM). In contrast, suspect and unexpected compounds can be tentatively identified using suspect screening (SS) and nontargeted screening (NTS). Rather than using RMs, these approaches rely on databases and cheminformatic tools. In SS, a molecular or structural formula from a target list serves as the starting point for identification. Conversely, NTS is entirely data-driven, operating without prior information about the compounds present. These strategies exploit information such as retention time (RT), ionized species, isotope patterns, or fragmentation profiles, which can be retrieved from databases, inferred, or calculated to support the identification of suspect list entries or candidate compounds proposed on the basis of NTS-detected signals.^5^

Developing TA, SS, or NTS methodologies for compound identification is a complex and time-consuming process that involves both experimental procedures and data analysis.^6^ In the experimental stage, targeted methods for the identification and quantification of multiclass compounds are routinely used in analytical laboratories. These methods are validated for hundreds or even thousands of chemically diverse compounds and can generate screening data during routine analysis. A common approach combines the Quick Easy Cheap Effective Rugged Safe (QuEChERS) extraction method^7^ with chromatographic separation and mass spectrometry using full scan (FS) or data-independent acquisition (DIA) modes.^8–11^ This setup produces far more data than required for routine TA, thereby enabling laboratories to extend targeted workflows to SS and NTS applications. Such flexibility enables laboratories analyzing diverse products to also detect suspect, unexpected, or unknown substances, thereby increasing the detection rate of contaminants.^12^

In general, data analysis for chemical identification relies on comparing identification properties with those of a reference, ideally a reference material (RM) analyzed under the same conditions. However, the limited availability of RMs, due to physical and cost constraints, often restricts this approach. In SS and NTS analyses, additional limitations arise from the lack of reference information such as RT or fragmentation mass spectra for suspects or nontargeted compounds. Although public and proprietary databases are continuously updated,^13–15^ their growth lags behind the emergence of new compounds of concern.

Identification properties can include quantitative measurements such as accurate mass, isotope patterns, RT, and fragmentation spectra; calculated values such as spectral similarity and mass defect; and qualitative descriptors such as ionization plausibility, volatility, lipophilicity, and methodological amenability.^6,16–20^ Combining multiple descriptors strengthens the identification hypothesis and recent software developments aim to incorporate as many of them as possible. However, many informatic tools, particularly vendor platforms commonly used in routine laboratories, do not cover the full range of descriptors or do not present their contributions in a way that allows analysts to evaluate them individually, which limits the interpretability of results. Most of these tools prioritize quantitative properties because they provide high-quality information.^19–21^ Nevertheless, as SS and NTS approaches become more common, qualitative evidence is gaining importance, as it can reinforce the identification and help reduce the chemical space explored by the analytical methodology.^6,22^

Machine learning (ML) has been increasingly applied to infer identification properties, mainly through individual tools that predict RT, ionization efficiency, compound amenability, and in silico fragmentation spectra, among others.^19,20,22,23^ Several strategies have been proposed to integrate multiple identification information for candidate compounds. For example, Woldegebriel^21^ developed a fully probabilistic Bayesian model to estimate the likelihood of candidate presence in routine screening, focused on quantitative parameters such as chromatographic properties of isotopologues, isotopic features of precursors and fragments, ionized species (e.g., adducts), and RT. Alygizakis et al.^19^ proposed an ML-based scoring system that combines several values (mass accuracy, retention index, isotopic fit, MS/MS fragments, spectral similarity, and molecular formula of fragments) to classify identifications as high or low confidence. Their model was trained on experimental data from multiple laboratories to assign scores to each property, improving the interpretability of identification results from HPLC-MS/MS analyses. However, this approach remains restricted to quantitative parameters and does not include qualitative ones useful for SS or NTS analysis. Similarly, Lestremau et al.^24^ developed an ML-based algorithm that integrates calculated properties such as RT, ion abundances, isotopic profiles, and fragmentation patterns at different energies, but it also focused exclusively on quantitative data.

In this work, we introduce an integrated framework for data analysis of a QuEChERS-HPLC-ESI-HRMS methodology employing variable data-independent acquisition (vDIA). The framework evaluates protonated and deprotonated species and incorporates a collection of machine learning models that infer analytical information when experimental evidence is unavailable. This capability increases its applicability to suspect and nontargeted screening workflows. Depending on the amount and quality of available evidence, the resulting identifications may correspond conceptually to Schymanski confidence Levels 1–3.

The framework builds on the analogy between chemical identification and quantitative analysis: in quantitative analysis, calibration models use one or more references to obtain measurement results, while in identification, RMs provide information that is compared with that of the analyte through a comparison model. This information serves as inputs to the comparison model and contributes to the overall identification, analogous to input quantities in a quantitative uncertainty budget. An uncertainty budget details the estimation of measurement uncertainty by specifying elements such as the measurement model, input quantities, their uncertainties, and probability distributions. The relative contribution of each input, defined for uncorrelated variables as the ratio of its variance to the combined output variance, can be visualized using a bar chart.^25–27^

From this perspective, the main challenge in identification lies in the limited availability of reference properties within the analytical methodology. To address this limitation, we developed ML models capable of inferring missing reference properties. This approach allows TA, SS, and NTS analyses to be addressed within a unified conceptual framework, defined by the amount and quality of available reference properties. We developed *Identificands*, a software platform that implements this framework by integrating inference models for multiple identification properties, comparing them with experimental data, and generating a quantitative estimate of confidence in the proposed identification, analogous to uncertainty budgets in quantitative metrology.

## Methods

### Theoretical Methods

Analytical samples can be described as a defined chemical space composed of matrix components and analytes. Within the analyte subspace, experimental measurements yield analytical properties that subdivide this space into overlapping or, ideally, nonoverlapping subsets of compounds. When both the number of properties and discriminatory power are sufficient, these subsets can be associated with specific compound identities within a defined level of uncertainty. Although individual properties may provide limited discriminatory power, their combination typically improves resolution in the analyte subspace and improves compound identification.^20,21,28^

In this process, quantitative properties (measurands)^25^ are generally preferred over qualitative ones (examinands).^29^ However, qualitative properties remain valuable, especially for identifying unexpected compounds that are not explicitly targeted by the analytical methodology.^6,30^ Both types of properties ultimately serve the same function: determining whether a compound fits the analytical criteria. For example, a candidate may comply or fail to comply with RT requirements, or may show sufficient or insufficient agreement with a mass fragmentation profile. To formalize this role, we introduce the term identificand, which encompasses both measurands and examinands obtained from an analytical methodology and used for compound identification (see the glossary of acronyms and key technical terms in the Supporting Information).

Given a set of identificands for an analyte and a candidate reference compound (RC), the analyte can be assigned to the RC when two conditions are satisfied: (i) each identificand agrees within its measurement uncertainty, and (ii) the identificand values differ from those of all other compounds. Together, these conditions define the general identification principles.^16^ RCs may originate from RMs analyzed in the laboratory or from compounds obtained through database searches or in silico structure prediction.^23,31–33^

Identification can be understood in analogy to quantification. In quantification, a calibration model is established using an RM and then applied to generate a measurement result.^25^ In identification, a comparison model and the identificand values of an RC are used to evaluate whether the analyte corresponds to the reference, yielding an identification result. For quantitative identificands, predefined metrics and acceptance criteria support categorical assignment. For qualitative identificands, the criterion is the existence or absence of a match with a defined category. Each categorical outcome has an associated probability, and the joint consideration of these probabilities provides a quantitative measure of identification confidence.^16,21,34^

In TA, the identificand values of a RC are obtained by measuring the corresponding RM with a defined analytical methodology. In SS analysis, identification is more complex because not all identificand values of an RC are known in advance. The same limitation applies to NTS workflows, where candidate compounds are proposed based on accurate mass and generally lack prior evaluation using the employed analytical method. When a RM is unavailable, identificand values may be retrieved from reference libraries that are compatible with the analytical methodology.^28^ However, if the methodology is not fully compatible with the library data, or if the library lacks identificand values for the compound of interest, reliable identification cannot be achieved. From this perspective, TA, SS, and NTS approaches differ mainly in the quality and prior availability of identificand information during data analysis.

Current screening methodologies for pesticide residues analysis are validated with hundreds of RMs across diverse matrices. Previous studies have shown that analytical results contain information that can be exploited by ML models to infer missing identificand values for RCs during data analysis, particularly for compounds outside the validated scope.^19,20,22^ Building on this concept, we developed an ML-enhanced QuEChERS-HPLC-ESI-HRMS Orbitrap methodology for pesticide residue analysis in fruits and vegetables, in accordance with the general principles of identification.

An identificand is qualitative when it employs a model to establish correspondence between a property and a candidate compound; it is quantitative when it uses a metric to assess metrological compatibility between the RC and the analyte property. Within this framework, six identificands associated with the analytical methodology were considered. These identificands were selected because they collectively capture the information generated across all stages of our previously developed and validated in-house workflow, including extraction, chromatographic separation, ionization, and detection. Four are qualitative: (i) methodological amenability, representing the likelihood that a compound can be successfully extracted via QuEChERS, separated chromatographically, and ionized by ESI; (ii) the quality of the extracted ion chromatogram (XIC) peak, assesses the morphological reliability of the signal to distinguish true chromatographic peaks from spurious noise^35^ (iii) the ion species formed, indicating the propensity of a compound to produce protonated or deprotonated ions;^36^ and (iv) the MS/MS fragmentation profile, which assesses the consistency of observed fragments with those expected for a candidate compound, rather than employing a metric for spectral comparison between an RC and the suspect or unknown analyte.^37–39^ The remaining two are quantitative: (v) the RT, which reflects the physicochemical behavior of the RC during chromatographic separation,^40–43^ and (vi) the isotopic pattern, which includes the monoisotopic mass and the distribution of isotopic peak intensities in the mass spectrum. Together, these properties provide a structured basis for retrieving and ranking candidate compounds through exact-mass or molecular-formula searches in reference libraries.^17,23^

The isotopic pattern—calculated from the assigned molecular structure—and the XIC peak quality are evaluated in all workflows for every detected analyte. In TA, identificand values are known for both the RC and the analyte, requiring only an assessment of their agreement. Conversely, candidate compounds in SS and NTS lack reference values; therefore, properties such as methodological amenability, ionized species type, MS/MS fragmentation profiles, and RT must be inferred (e.g., via ML models). In SS, this inference applies only to missing identificands, whereas NTS requires inferring all such properties for every candidate structure consistent with the detected accurate mass.

### Quantitative Identificands

To evaluate the similarity between a reference compound R and an analyte signal A quantitative metrics are required. For the RT identificand $\left(I_{\mathrm{RT}}\right)$ , similarity can be expressed as $s_{I_{\mathrm{RT}}}=t_{A}-t_{R}$ , where $t_{A}$ and $t_{R}$ are the analyte and reference RTs, respectively. The $t_{R}$ value is experimentally derived in TA, whereas in SS and NTS, it is inferred from the assigned molecular structure via an ML model when reference standards are absent.

For an isotopic pattern identificand $\left(I_{\mathrm{IP}}\right)$ , an isotopic pattern is defined by two *n*-dimensional vectors: **m** for m/z values and **a** for relative isotopic abundances. Given an analyte isotopic pattern $I_{{\mathrm{IP}}_{A}}=\left(m_{A},a_{A}\right)$ and a reference isotopic pattern $I_{{\mathrm{IP}}_{R}}=\left(m_{R},a_{R}\right)$ , similarity can be quantified as

$$s_{I_{\mathrm{IP}}}=\sqrt{e_{m}^{2}+e_{a}^{2}}$$ 1

where *e*_*m*_ and *e_a_* are the relative errors between the analyte and reference vectors m/z and isotopic relative abundance dimensions, respectively. The error in dimension v=m,a is calculated as

2 $$e_{v}=\frac{V_{A}\cdot V_{R}-V_{R}\cdot V_{R}}{d_{v}\cdot V_{R}}$$

with $v_{A}$ and $v_{R}$ representing the analyte and reference values, and $d_{v}$ is an accepted deviation vector.

Because the similarity metrics for RT and isotopic pattern are random variables (*S*), assuming $S∼N\left(\mu_{S,}\sigma_{S}^{2}\right)$ , their probability of matching a reference property can be expressed as^44^

3 $$p\left({\mathrm{match}}_{s}|R\right)=\frac{P\left\{S\leq s||S-\mu_{S}|\leq k\sigma_{S},|S|\leq c_{S}\right\}}{P\{|S-\mu_{S}|\geq k\sigma_{S}\}}$$

where *P*{·} denotes the probability under a normal distribution, *c*_*S*_ is the acceptance criterion, and *k* is a coverage factor defining the confidence interval of *S* (e.g., k≈1.96 for a 95% confidence level in a normal distribution).

In targeted approaches, $t_{R}$ is known experimentally with low uncertainty. By contrast, the error and uncertainty in the ML-inferred RT are higher in the SS and NTS approaches. To account for these differences between experimental determined values in TA and ML-inferred values in SS and NTS, the probability obtained from eq 3 can be weighted with

4 $$w_{\mathrm{RT}}=\frac{1}{1+\log_{10}\max\left(1,U_{\mathrm{RT,ref}}/U_{\mathrm{RT,measured}}\right)}$$

where *U*_RT_,_ref_ is the uncertainty for the reference RT, determined either experimentally or by ML inference.

### Qualitative Identificands

Qualitative identificands have different levels of relevance during data analysis. The XIC peak quality identificand $\left(I_{\mathrm{XQ}}\right)$ and the MS/MS fragmentation profile identificand $\left(I_{\mathrm{MS2}}\right)$ are important in both target and NT approaches. In contrast, compound amenability $\left(I_{A}\right)$ and type of ionized species $\left(I_{\mathrm{IS}}\right)$ are more relevant for NT approaches. The $I_{\mathrm{MS2}}$ is particularly important for reducing the number of candidate compounds associated with a given analytical signal,^28^ while $I_{\mathrm{XQ}}$ is essential for lowering the false-positive rate and prioritizing signals for further analysis.^35^ $I_{A}$ and $I_{\mathrm{IS}}$ are especially relevant for assigning analytical signals to molecular structures, as they help narrow the identifiable chemical space in NT approaches.^30,36^ This is important for compounds not previously assayed with the analytical methodology, and it is critical in automated identification workflows such as the one presented here.

In this work, Random Forest (RF) classifiers^45,46^ were implemented to infer the category and associated probability for the identificands $I_{\mathrm{XQ}}$ , $I_{A}$ , and $I_{\mathrm{IS}}$ . While the $I_{\mathrm{XQ}}$ value is derived from the XIC of the detected analytical signal, $I_{A}$ and $I_{\mathrm{IS}}$ are inferred from the molecular structure of each RC, represented as Simplified Molecular Input Line Entry System (SMILES) strings.^47^ These SMILES-based RF models predict method amenability and the specific ionized species formed (e.g., [M + H]^+^ or [M - H]^−^), which indirectly dictates the required detection mode (ESI+ or ESI−).

For $I_{\mathrm{MS2}}$ , *MetFrag* ^48^ was used to assess whether the observed fragment ions are attributable to a candidate RC without requiring MS/MS spectral libraries. While this approach applies to SS and NTS workflows—where properties are inferred from the candidate RC—TA relies on known experimental fragments. The approach uses a cleaned fragmentation mass spectrum and one or more candidate RCs as inputs to *MetFrag*. The software annotates the observed fragments, and when annotation is successful, the annotated ions are used to calculate a probability for $I_{\mathrm{MS2}}$ . This procedure was applied to Orbitrap mass spectrometry data.

To obtain the cleaned fragmentation mass spectrum, the procedure takes advantage of the correlation between the FS-XIC of nonfragmented ions and the XICs of product ions of a given analyte. The FS-XIC peak can therefore be used as a template to identify candidate ions in the MS/MS spectrum of R . For normalized XICs peaks represented as *n*-dimensional intensity vectors **I**_MS2,*i*_ and **I**_FS_, the similarity between the FS peak and the *i*-th MS/MS XIC peak is calculated as

5 $$s_{\mathrm{FS}‐\mathrm{MS2},i}=\frac{||I_{\mathrm{FS}}-{I_{\mathrm{MS2,}i}||}^{2}}{n\sigma_{d}^{2}}$$

where *σ*_d_ denotes the accepted intensity deviation threshold, *n* is the number of points in the *i*-th MS/MS peak within the time interval defined by the FS peak, and the components of **I**_FS_ are reduced to *n* points by sampling the FS peak intensities at time points closest to those of the MS/MS peak. The probability associated with $s_{\mathrm{FS}‐\mathrm{MS2}}$ is then calculated as

$$p\left(s_{\mathrm{FS}‐\mathrm{MS2,}i}\right)=1-F_{S}\left(s_{\mathrm{FS}‐\mathrm{MS2,}i},n,v_{d}\right)$$ 6

where *F*_*S*_ is the cumulative probability of an F-distribution. The cleaned fragmentation spectrum is obtained from MS/MS signals with $p\left(s_{\mathrm{FS}‐\mathrm{MS2}}\right)\geq p_{\mathrm{Threshold}}$ . For *m MetFrag*-annotated ions, the probability of $I_{\mathrm{MS2}}$ given R is calculated as

7 $$p\left(I_{\mathrm{MS2}}|R\right)=\frac{w}{m}\sum_{i=1}^{m}p\left(s_{\mathrm{FS}‐\mathrm{MS2},i}\right)$$

where *w* is a weight derived from the *MetFrag* score for a given candidate compound.

### Identification Confidence

Given a set of identificands for the analytical methodology $\left\{I_{i}\right\}\left(i=1,...,n\right)$ and a candidate R , the probability of identification can be expressed as

8 $$p\left(R|I_{1},...,I_{n}\right)=\frac{p\left(R\right)p\left(I_{1},...,I_{n}|R\right)}{p\left(R\right)p\left(I_{1},...,I_{n}|R\right)+p\left(\neg R\right)p\left(I_{1},...,I_{n}|\neg R\right)}$$

Woldegebriel studied the calculation of $p\left(R|I_{1},...,I_{n}\right)$ for some quantitative identificands.^21^ However, as the number of identificands increases, the calculation becomes more complex, particularly when they are not independent. Because the complete space of RCs is unknown, a simplified approach can be applied by assuming independence among the selected identificands. Under this assumption, identification confidence can be related to the Euclidean distance between six-component identificand probability vectors

9 $$d=\sqrt{\left(p-q\right)\cdot \left(p-q\right)}$$

where $p=\left(pI_{A},pI_{\mathrm{RT}},pI_{\mathrm{XQ}},pI_{\mathrm{IS}},pI_{\mathrm{IP}},pI_{\mathrm{MS2}}\right)$ and **q = 1**. The value *p*_identification_ = 1–*d/√n* for *n* identificands can be interpreted as a measure of identification confidence. The contribution of a particular identificand to the identification confidence can be assessed by calculating the percent change in *p*_identification_ when the probability of that identificand is set to 1, while keeping all other identificand probabilities constant.

The definition, calculation, and combination of these identificands, alongside the estimation of their contributions, provide a framework analogous to the uncertainty budget in quantitative analysis.^25^ The specific parameters and decision thresholds used for each identificand are detailed in Table S1 of the Supporting Information.

### Computational Methods

#### Machine Learning Models

ML models improve SS and NTS workflows by predicting identificand values when physical reference materials are unavailable. In SS, ML infers specific missing properties for user-provided suspect compounds. Conversely, in NTS, data-driven candidates are proposed, requiring ML to automatically infer all necessary identificand values without user guidance.

Every workflow requires identificand values for the analyte and RC. Individual ML models infer missing properties to determine $I_{\mathrm{XQ}}$ , $I_{A}$ , $I_{\mathrm{IS}}$ , and $I_{\mathrm{RT}}$ as needed. To infer $I_{\mathrm{XQ}}$ , a classifier uses the detected mass XIC to predict a quality category and its probability. To determine $I_{A}$ , $I_{\mathrm{IS}}$ , and $I_{\mathrm{RT}}$ , molecular descriptors derived from the SMILES string are used to derive the model features. To obtain $I_{A}$ , a classifier predicts amenable or not amenable probabilities; to obtain $I_{\mathrm{IS}}$ , another infers the ionized species type and probability; and to obtain $I_{\mathrm{RT}}$ , a regressor predicts the RT. RF was used for classification and Extreme Gradient Boosting (XGBoost) for regression.^46,49^ Both algorithms handle nonlinear, high-dimensional data efficiently and are widely applied in chemical identification due to their predictive accuracy and computational performance. Previous studies have consistently ranked these models among the best-performing methods for predicting molecular properties such as ionizability and RT.^19,31,36,50–52^

#### Input Features

For models targeting $I_{A}$ , $I_{\mathrm{IS}}$ , and $I_{\mathrm{RT}}$ , 217 molecular descriptors were derived from SMILES strings using *RDKit* ^53^ and *Mordred*.^54^ Numerical variables were transformed using the Yeo-Johnson power method;^55^ missing values were imputed using the median for continuous variables and zero for integers. Dimensionality reduction through Principal Component Analysis (PCA) retained components explaining 95% of the total variance. Consequently, 92, 90, and 67 principal components were used as input features to infer $I_{A}$ , $I_{\mathrm{IS}}$ , and $I_{\mathrm{RT}}$ , respectively.

Input features to infer $I_{\mathrm{XQ}}$ consist of 864 components derived from a Histogram of Oriented Gradients (HOG),^56^ computed from a 5625-pixel image representing the XIC of the detected analyte.

#### Optimization

RF hyperparameters were optimized for the number of trees, the number of features considered at each split, and cost-complexity pruning. XGBoost hyperparameters, including the number of estimators, learning rate, subsample ratio of training instances, maximum tree depth, column subsampling during tree construction, and the minimum sum of instance weight needed in a child, were optimized using the Hyperopt tool.^57^

#### Training Data Set

The model for $I_{A}$ was implemented as a binary RF classifier using categories amenable and unamenable. The training set comprised 3819 compounds: 889 positive instances obtained from Datapool and in-house measurements (pesticides validated for QuEChERS-LC-ESI-MS) and 2930 negative examples.^36,58^ The positive instances cover a broad hydrophobicity range, with calculated octanol–water partition coefficients spanning from −1.25 to 8.52. Consistent with this range, these compounds are distributed across the entire chromatographic gradient. The negative instances include highly polar compounds, highly hydrophobic compounds, and nonionizable species that are not amenable to QuEChERS extraction, liquid chromatography, or electrospray ionization. Therefore, the model is expected to perform best for compounds falling within the physicochemical space represented by these classes.

The model for $I_{\mathrm{IS}}$ was implemented as a binary multilabel RF classifier targeting the species [M + H]^+^ and [M – H]^−^, with each label categorized as observed or unobserved. The training set included 10469 compounds, comprising 7641 positive cases for protonated species and 4661 for deprotonated species. Ionized species data were compiled from in-house measurements and the MassBank database.^14^

The model for $I_{\mathrm{XQ}}$ was implemented as a multiclass RF image classifier with three categories: noise, problematic, and acceptable. The training set comprised 6512 XICs, including 1192 noisy, 677 chromatographically problematic (e.g., multipeak or unstable profiles), and 4643 acceptable signals (including high-quality, split, or overlapping peaks). Data were obtained from in-house experiments.

The model for $I_{\mathrm{RT}}$ was implemented as an XGBoost regressor trained on 591 compounds using in-house experimental data from RMs prepared in solvent.

All data sets were manually curated and split into training/validation (80%) and test (20%) subsets. To prevent data leakage when combining data from multiple sources, duplicate compounds were removed based on their SMILES representation before data set splitting. For the amenability and RT models, the data were stratified into ten bins according to the octanol–water partition coefficient to preserve the distribution of class ratios across the subsets. For the ionized species model, iterative stratification was used to maintain label distributions across the splits.^59^ The data sets and scripts used for model training and retraining are publicly available at: https://github.com/sagm-co/msLabAIssistant/tree/main/ML-Models.

#### Model Assessment

To address class imbalance in the classification tasks, performance was evaluated using the Matthews correlation coefficient (MCC), the area under the precision-recall curve (AUC-PRC), and the area under the multiclass classification curve (AUC-MCP);^60^ accuracy, recall, and precision were also calculated. For regression, performance was assessed using the mean absolute error (MAE), root-mean-square error (RMSE), and coefficient of determination (*R* ^2^).^45,46^ Training performance was validated through 10-fold random permutation cross-validation (shuffle and split). All performance metrics are reported using fixed decision thresholds: 0.5 for binary models and the highest predicted probability for the multiclass model.

#### Software and Hardware

All software development and calculations were carried out on a Dell laptop equipped with an AMD Ryzen 7 5800H processor (4.5 GHz, 8 cores, 2 threads per core), 16 GB RAM, and an NVIDIA GeForce RTX 3050Ti graphics card. The operating system was Linux/Ubuntu 22.04.4 LTS.

The software was implemented in Python 3.10 using scikit-learn 1.3.2 ^61^ and TensorFlow 2.15.0.^62^ The application integrates open-source tools via wrappers, including *RDKit*,^53^ *Mordred*,^54^ *MetFrag*,^48^ *BrainPy*,^63^ and the O’leary’s Python wavelet library.^64^ Scikit-multilearn was used to split training sets for multilabel classification.^59^ Vendor libraries were employed to directly access and process Thermo Scientific raw files, enabling automatic batch processing without external conversion tools.^65^ For performance evaluation, *Tracefinder* 4.1, *Compound Discoverer* 3.1 (Thermo Fisher Scientific), and *MZmine* 4.5.0 ^66^ were used. As the instrumental acquisition was performed in positive polarity, data analysis was restricted to protonated species for simplicity.

#### NTS Chemical Space

A contaminants database was downloaded from the NORMAN server,^15^ curated to remove counterions, and validated for SMILES integrity using *RDKit*.^53^ Based on this, a contaminants chemical space reference library (CRL) was constructed by combining the curated NORMAN data set with an in-house contaminants list. The CRL contains 96518 entries and includes the following fields: compound name, canonical SMILES, InChIKey, molecular formula, and monoisotopic mass for the neutral compound. This CRL was used to restrict chemical searches during NTS data analysis.

#### Software Development and Workflows Logic

*Identificands* is a software system that integrates workflow logic for processing raw data with the identificand concepts described in Section Theoretical Methods. The workflow was implemented using an Object-Oriented Paradigm and is graphically illustrated in Figure 1. The ML models for identificand inference were developed using data generated from our in-house validated QuEChERS-HPLC-ESI-HRMS methodology for pesticide residue analysis in fruits and vegetables.^67^ Application of the workflow to other analytical platforms requires retraining the amenability, RT, and/or ionized species models using data generated under the corresponding analytical conditions. In particular, RT models must be retrained when different chromatographic modes (e.g., reversed-phase or HILIC) or gradient programs are employed. Once trained, additional ML models can be readily integrated into the Identificands framework.

**Figure 1:**
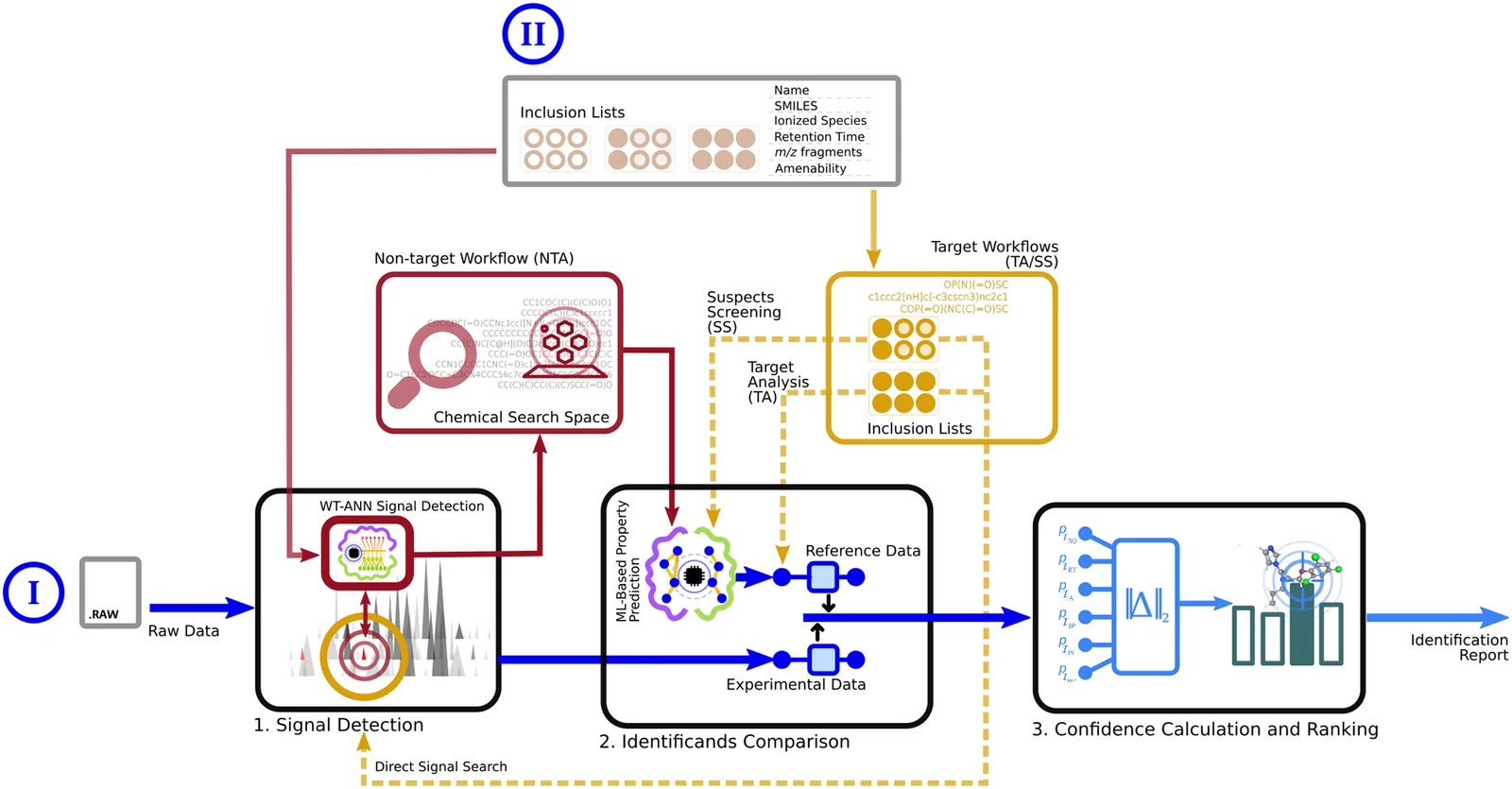
Data analysis workflow for the identification of chemical compounds using *Identificands*. Information flow in TA and SS signal detection is indicated by dashed arrows, while solid red arrows represent information flow in NT. Blue solid arrows denote the common information flow shared by both target and NT approaches. Solid and open circles in the inclusion lists represent provided and missing values, respectively. An NTS workflow begins with all fields in the list empty.

The software requires a raw data file and an inclusion list containing five parameters: SMILES, type of ionized species (protonated, deprotonated, or adduct), RT, *m*/*z* of MS/MS fragments, and information on compound amenability to the analytical methodology. These parameters can be provided in full or in part, depending on the available information. The completeness of the input determines which identification approach is applied by the software.

The software incorporates independent targeted (TA/SS) and NTS workflows, selected based on the data provided in the inclusion list. Specific targeted workflows are further determined by the inputs provided. For instance, a TA approach requires all parameters, whereas a SS approach requires at least the compound SMILES. Missing input data are inferred using the implemented ML models. The targeted and NTS workflows share three main steps, as shown in Figure 1: (1) signal detection, (2) identificands comparison, and (3) calculation and ranking of identification confidence.

The targeted workflows comprises three main steps. First, an FS-XIC is obtained for the monoisotopic mass of the specified or inferred ionized species (default *m*/*z* tolerance: 5 ppm). The XIC is then evaluated using the RF model trained for peak image recognition. If the chromatographic signal is classified as acceptable, it is processed further. Second, probabilities for identificands are calculated, and compounds with probability below a defined threshold (0.5 by default) are discarded. Finally, identification confidence is calculated and exported as a tab-delimited file. This output includes the probability associated with each identificand, the overall confidence score, and the individual effect of each identificand on the final combined confidence for each target or suspect compound.

The NTS workflow comprises six general steps, four of which correspond to signal detection. First, for peak picking, the software applies an algorithm based on probable regions (PR) of isotopic patterns in the *m*/*z* dimension, similar to the approach used in *XCMS*.^68^ In our case, the Wavelet Transform (WT) is applied solely to locate PRs.^69^ Within these noisy PRs, IPs are identified using a custom-trained artificial neural network (ANN). The WT-ANN combination acts as a denoising filter. Second, when the ANN recognizes an IP, each isotopic signal is labeled, and the FS-XIC corresponding to the monoisotopic signal is obtained (default *m*/*z* tolerance: 5 ppm).^70^ The XIC is then evaluated for peak quality using the RF model trained for peak image recognition. Signal features from peaks classified as acceptable are retained for subsequent processing. Third, for the retained features, ionized species corresponding to the same compound are grouped and saved as an unknown analytical signal for further analysis. Fourth, for each unknown analytical signal, the neutral monoisotopic mass is searched in the CRL to obtain candidate compounds. Fifth, SMILES of candidate compounds are processed using ML inference models to generate identificands probabilities. Finally, the confidence identification is calculated, and results are reported in a tab-delimited file. This output contains the same data fields as the targeted workflows for the candidate with the highest confidence score. Candidates with lower scores are reported in a separate tab-delimited file.

#### Workflows Performance Evaluation

Performance assessment focused on the structural identification accuracy of the data-processing workflows rather than a full analytical method validation. Identificands was benchmarked against *TraceFinder* for suspect screening (SS), and against *Compound Discoverer* and *MZmine* for nontargeted screening (NTS). Software selection was based on the widespread adoption of these platforms within the routine pesticide residue and food-safety analytical community rather than on compatibility with a specific acquisition strategy. *TraceFinder* and *Compound Discoverer* represent commonly used proprietary tools, whereas *MZmine* was selected as a widely adopted open-source alternative. Additional comparisons with platforms specifically developed for DIA data analysis, including *XCMS* ^68^ and *MS-DIAL* ^71^ would provide valuable benchmarks for future evaluations.

To ensure comparability and reflect typical usage, all platforms were executed with default parameters, except for harmonized *m*/*z* tolerances, intensity thresholds, and RT tolerances (Table S2). Because reference RTs are generally unavailable in SS and NTS workflows, they were not provided to the algorithms as prior information; instead, observed RTs were evaluated postprocessing. All analyses were conducted in ESI+ mode, targeting [M + H]^+^ ions.

To decouple algorithmic performance from instrumental effects (e.g., ion suppression and matrix effects), sensitivity was evaluated against a consensus reference set (CRS). For each matrix (avocado, lettuce, orange, and papaya) and concentration level (0.5, 1, and 10 ng/mL), the CRS was defined as the union of all true-positive identifications confirmed by *TraceFinder* and *Identificands* during SS and TA. Compounds were confirmed based on mass error (≤5 ppm), RT tolerance (±0.1 min), and the presence of MS/MS fragments. Algorithmic sensitivity was calculated as the proportion of CRS compounds correctly identified by each workflow. Absolute sensitivity was evaluated relative to the total number of spiked validation compounds.

False positive (FP) counts were determined using unspiked matrix extracts by counting compounds reported as identified but absent in the matrix blank extract. Additionally, for NTS workflows, the annotation burden—defined as the number of annotated compounds requiring manual confirmation—was included as a secondary metric. Details of the statistical modeling are provided in Section S1 of the Supporting Information.

## Experimental Methods

### Chemicals and Reagents

A suite of 778 compounds was prepared using two working solutions. Working Solution A (745 compounds, 1 μg/mL) was prepared by pooling five 5 μg/mL stock mixtures from the Smart Solutions v700 PestiMix kit (Dr. Ehrenstorfer-LGC, North Charleston, SC, USA). Working Solution B (33 compounds, 10 μg/mL in LC-MS grade acetonitrile) was prepared from individual 500 μg/mL stock solutions of high-purity standards (purchased from LGC and Merck; Table S3). Prior to experiments, Solutions A and B were diluted in LC-MS grade acetonitrile (ACN) to prepare intermediate working solutions at 25 ng/mL and 250 ng/mL. Depending on the required final concentration, these intermediate solutions were independently added to blank matrix extracts to prepare the final injectable solutions at 0.5, 1.0, and 10 ng/mL for each compound. Additionally, atrazine-d5 (99.0%) and fenthion-d6 (99.0%) (Merck KGaA, Darmstadt, Germany) were prepared at 10 μg/mL in ACN and used as surrogate standards for extraction quality control. Triphenylphosphate (TPP, 99%; Dr. Ehrenstorfer-LGC, Augsburg, Germany) was prepared at 10 ng/mL in methanol/water (50:50, v/v) and used as an internal standard for instrumental quality control. All solutions were stored at −20 °C.

Of the total compounds, 594 were confirmed using the complete QuEChERS-HPLC-ESI-HRMS-Orbitrap methodology in positive ionization mode.^67^ The remaining 184 compounds were excluded from the validation set because neither precursor nor product ions were detected under the analytical conditions employed. Identification criteria required the detection of the protonated precursor ion and at least two coeluting fragment ions, all with a mass error of ≤ 5 ppm. These 594 compounds constituted the validation set. The complete list of compounds is available on Zenodo.

Reagents for sample preparations using the QuEChERS approach were obtained from commercial suppliers: trisodium citrate dihydrate (≥99.0%), sodium citrate dibasic sesquihydrate (≥99.0%), and anhydrous magnesium sulfate (≥99.5%) from Sigma-Aldrich (St. Louis, MO, USA); sodium chloride (≥99.5%) from J. T Baker (USA), and primary secondary amine (PSA, propylethylenediamine) sorbent from Agilent Technologies (USA). High-purity water (18.2 MΩ·cm) was produced and 0.2 μm-filtered using a Direct Q water purification system (Merck, Darmstadt, Germany). ACN (HPLC gradient grade, ≥ 99.0%), LC-MS-grade ACN, formic acid, and ammonium formate were obtained from Scharlau (Barcelona, Spain). Methanol (hyper-grade for LC-MS, ≥ 99.9%) was purchased from Sigma-Aldrich (St. Louis, MO, USA).

### Sample Preparation and Chemical Analysis

The matrices used to evaluate software performance were orange, papaya, lettuce, and avocado, representing a range of analytical challenges due to differences in acidity and fat content. Matrix extracts were fortified at 0.5, 1.0, and 10 ng/mL. These vial concentrations correspond to 0.005, 0.01, and 0.1 mg/kg in the original sample matrix, respectively. A blind assay was also conducted on five samples, which were independently analyzed by a second analyst using a different quantitative method. The blind assay included matrices of pineapple, chia seeds, papaya, and tomato.

Sample preparation followed European Commission Regulation 752/2014 (European Commission, 2014). Approximately 1 kg of each frozen product (−20 °C) was homogenized for 5 min, and the processed material was stored at −20 °C until extraction. Analytical portions of 10 g were used for each analysis. Samples were extracted following a European QuEChERS procedure.^72^ For the blind assay, 100 ng of atrazine-d5 and 600 ng of fenthion-d6 were added as surrogate standards prior to extraction. After extraction and cleanup, 70 μL of the final extract were used to prepare the injection vial. TPP was employed as an internal standard for instrumental quality control. Blank matrix and solvent blanks were run before each matrix group. The blank matrix and solvent blank were subtracted from the performance extracts and blind assay samples, respectively. Performance extracts were measured using an in-house method previously validated according to SANTE guidelines, employing vDIA.^67^ This approach provides comprehensive nontargeted data acquisition with high sensitivity and reproducibility, bypassing the precursor-selection limitations inherent to data-dependent acquisition (DDA). Although vDIA generates complex MS/MS spectra that complicate traditional identification, our framework mitigates this ambiguity by evaluating fragmentation consistency alongside multiple identificands.

Blind samples were quantified independently using an in-house SANTE-validated and ISO/IEC 17025-accredited methodology. The quantitative method used the same extraction procedure but employed a distinct chromatographic separation and an FS-parallel reaction monitoring (PRM) acquisition. Identification results were compared after both analysts submitted their independent reports.

### Instrumentation

Chromatographic separation and mass spectrometry analysis were performed using a DionexTM Ultimate 3000 UHPLC system (Thermo Scientific, San Jose, CA, USA) coupled to a Q-Exactive Focus mass spectrometer equipped with a HESI ionization source (Thermo Scientific, Bremen, Germany). Mobile phase A consisted of 98% water and 2% MeOH, while mobile phase B consisted of 98% MeOH and 2% water. Both phases contained 5 mM ammonium formate and 0.1% formic acid as additives to enhance ionization. The elution gradient is shown in Figure 3b.

Chromatographic separation was carried out on an Accucore aQ C18 UHPLC column (Thermo Scientific, San Jose, CA, USA) with dimensions of 100 mm × 2.1 mm, and a particle size of 2.6 μm. Mass spectra were acquired in FS and variable data-independent acquisition (vDIA) modes over an *m*/*z* range of 140–900. Data were recorded in centroid mode. The resolution at *m*/*z* = 200 was 70000 for FS mode and 35000 for vDIA mode. Five vDIA segments were used: [140,216], [215,266], [265,316], [315,391], and [390,900] *m*/*z*. The HRMS-Orbitrap was calibrated in positive mode for the *m*/*z* scale using the manufacturer’s calibration solution. Complete experimental details are available in a previous publication.

## Results and Discussion

### Inference Models

Tables 1 and 2 summarize the performance metrics of the developed models. Metrics were obtained from ten random cross-validation permutations during training and five random 50/50 splits for testing. Classifier uncertainties remained below 4%, indicating consistent agreement and stable performance across different training and validation subsets. Training and testing results showed overall concordance, confirming that the models perform consistently on unseen data. Although further hyperparameter optimization and additional training data could improve performance, the observed results are comparable to those reported in previous studies.^36,41^ Therefore, the models exhibit sufficient robustness to be applied within the identification framework proposed in this work.

**Table 1:** Performance of the RF Classifier Evaluated by Random Permutation Cross-Validation during Training^b^

		Ionized Species		XIC-peak Quality
Performance Metric	Compound Amenability	[M + H]^+^	[M – H]^−^	Acceptable
Train (Cross-Validation)				
Accuracy	0.928 ± 0.007	0.887 ± 0.005	0.828 ± 0.005	0.868 ± 0.006
Recall	0.779 ± 0.021	0.961 ± 0.005	0.793 ± 0.009	0.961 ± 0.004
Precision	0.895 ± 0.022	0.893 ± 0.004	0.816 ± 0.008	0.887 ± 0.008
MCC	0.790 ± 0.020	0.702 ± 0.013	0.651 ± 0.009	0.690 ± 0.016
AUC(PRC)/AUC(MCP)^a^	0.915 ± 0.006	0.973 ± 0.003	0.890 ± 0.005	0.617 ± 0.004
Test				
Accuracy	0.923 ± 0.012	0.906 ± 0.002	0.838 ± 0.005	0.882 ± 0.003
Recall	0.767 ± 0.030	0.941 ± 0.002	0.760 ± 0.011	0.958 ± 0.002
Precision	0.898 ± 0.033	0.931 ± 0.003	0.861 ± 0.006	0.908 ± 0.002
MCC	0.782 ± 0.037	0.761 ± 0.005	0.673 ± 0.010	0.726 ± 0.008
AUC(PRC)/AUC(MCP)^a^	0.925 ± 0.019	0.980 ± 0.001	0.901 ± 0.001	0.627 ± 0.003

a AUC­(PRC) corresponds to the binary classifiers for methodological amenability and protonated/deprotonated ionic species. AUC­(MCP) corresponds to the multiclass classifier for chromatographic signal quality.

b Classifiers correspond to compound methodology amenability, feasibility of generating protonated or deprotonated species, and chromatographic signal quality. Results are reported with precision uncertainties at the 95% confidence level using the *t*-student distribution.

**Table 2:** Performance of the XGBoost Retention Time Predictor^a^

Performance Metric	Train (cross-validation)	Test
Mean absolute error (MAE)/min	0.749 ± 0.108	0.718 ± 0.039
Root-mean-square error (RMSE)/min	0.978 ± 0.140	0.949 ± 0.061
Coefficient of determination R^2^	0.666 ± 0.062	0.715 ± 0.019

a Results are reported with precision uncertainties at the 95% confidence level using a *t*-student distribution.

Results presented in Table 1 and Figure 2 show that, for the binary classifiers, the AUC­(PRC) values for testing exceeded the proportion of positive instances for each model (0.233 for amenability, 0.730 for protonated species, and 0.445 for deprotonated species). For the multiclass classifier of chromatographic signal quality, the AUC­(MCP) surpassed the expected value for a random three-class classifier (0.342), as indicated by the dashed red line in Figure 2d.2. These findings indicate that all classifiers perform significantly better than chance.

**Figure 2:**
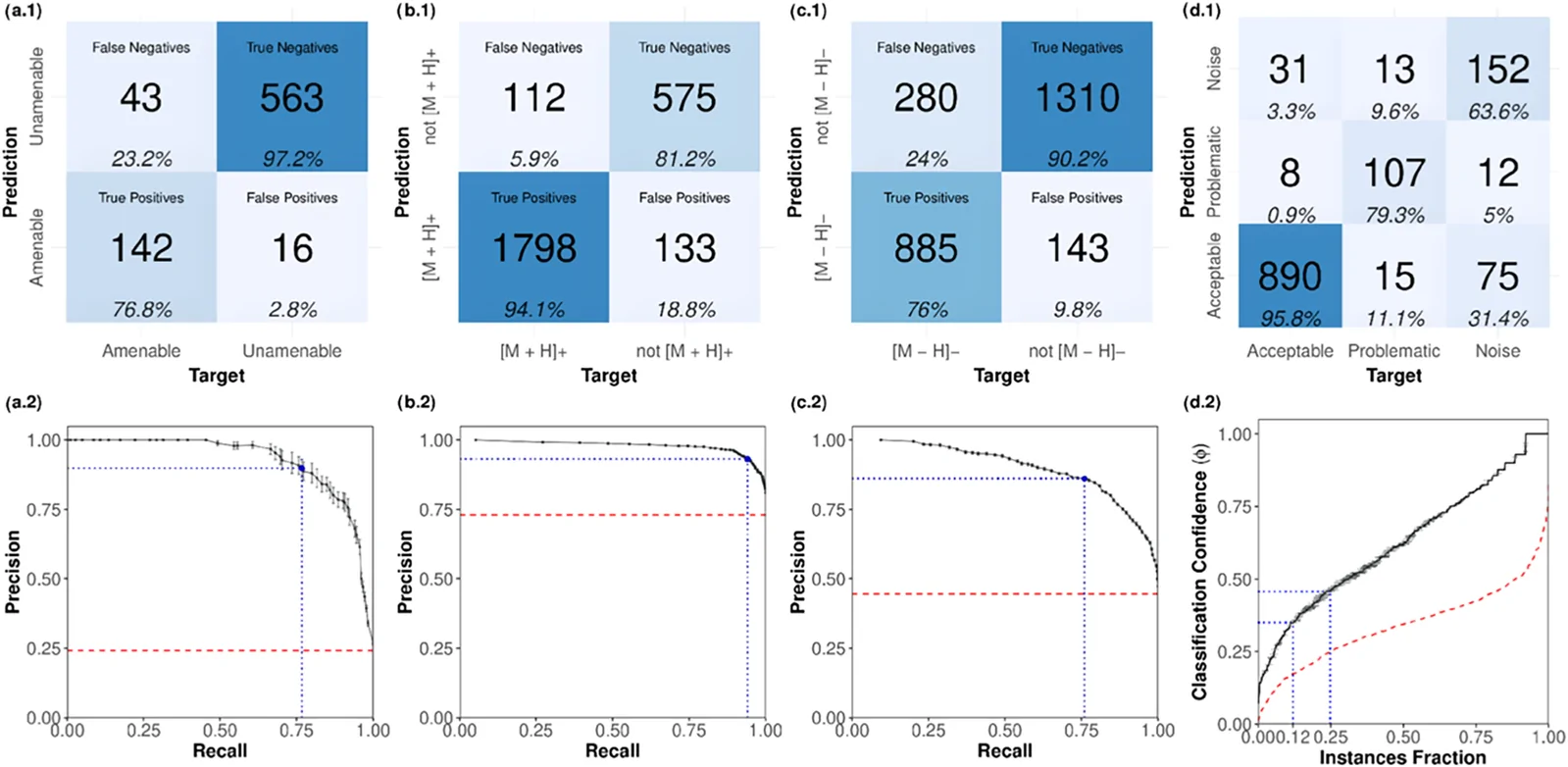
Confusion matrix (top) and precision-recall/multiclass performance curves (bottom) for test assessment: (a) binary classifier for methodology amenability; (b-c) multilabel classifiers for the feasibility of protonated and deprotonated species during ionization; and (d) multiclass classifier for chromatographic signal quality. Matrices display absolute counts and percentages. For the binary classifiers (a-c), the quadrants are labeled as True Positive, False Positive, True Negative, and False Negative. Vertical and horizontal dotted lines indicate operational points at a 0.5 threshold for binary classifiers or the maximum-probability criterion for the multiclass classifier. Dashed lines represent chance performance, and the dashed curve in (d.2) corresponds to a random three-class classifier. Error bars denote 95% confidence intervals calculated using the Student’s t distribution.

For the multiclass classifier, 75% of instances were correctly classified with a confidence of 0.459 or higher, while 12–25% were correctly classified with lower confidence (0.350–0.459). Approximately 12% of instances were misclassified, corresponding to an overall accuracy of 0.882. Regarding the MCC, all classifiers exhibited positive values (lower MCC = 0.651 ± 0.009), confirming robust performance in correctly classifying both positive and negative instances.

Table 2 and Figure 3 summarize the performance of the retention time ML model. The mean absolute error (MAE) for testing was 0.718 ± 0.039 min with an *R* ^*2*^ value of 0.715 ± 0.019. Similar to the classifier models, the regression model showed consistent performance between training and testing, with uncertainties of up to 0.14 min for MAE and RMSE, indicating moderate variability across cross-validation and test instances. Examination of the model error distribution (Figure 3) revealed that 73% of test instances (*n* = 119) exhibited errors below ± 1 min. However, a few compounds displayed substantial negative bias (e.g., dodemorph with −3.7 min and proquinazid with −2.6 min). This bias may result from limited training data at high retention times; with few examples in this late-elution range, the XGBoost model lacks sufficient data to capture the specific physicochemical interactions governing retention.

**Figure 3:**
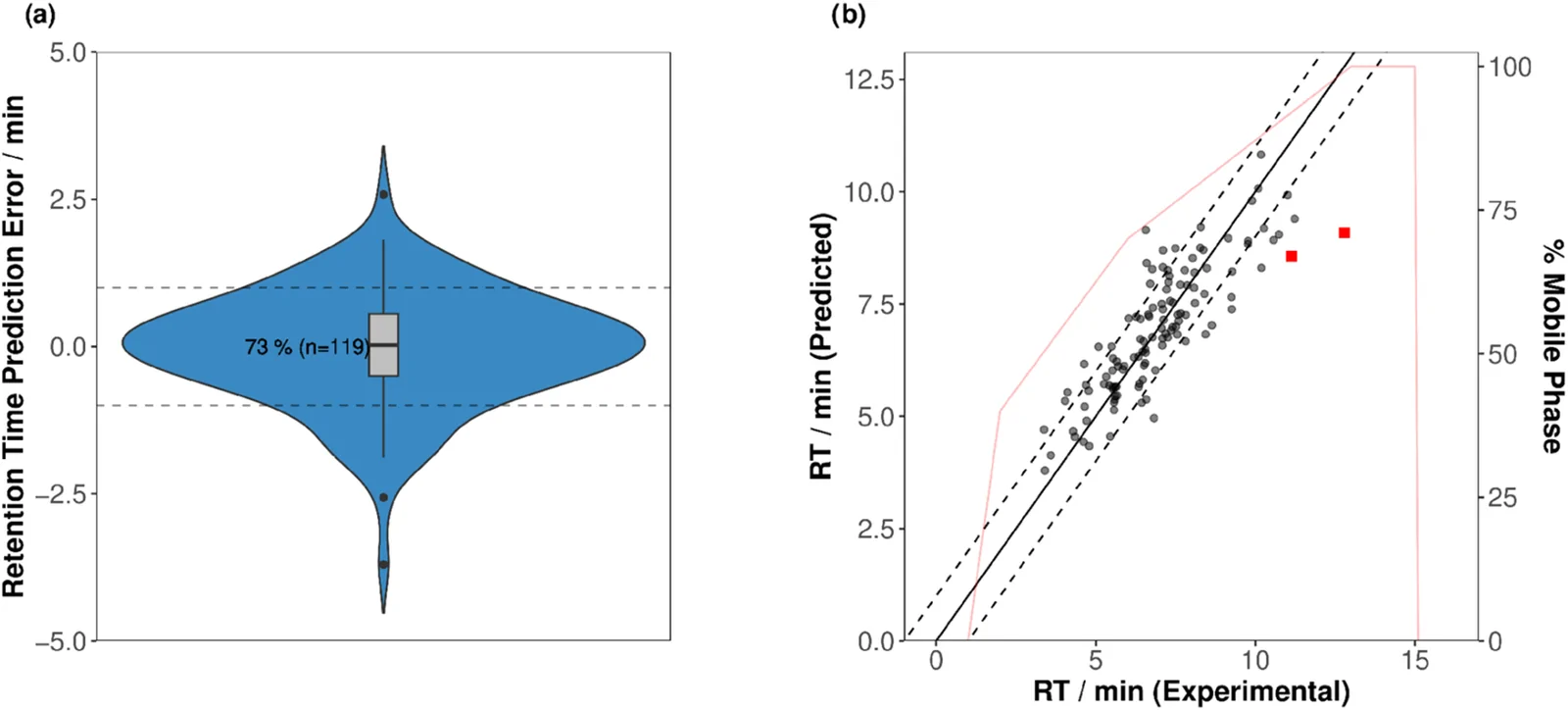
(a) Error distribution of the retention time prediction model. (b) Predicted versus experimental retention times for testing instances, with dashed lines indicating ± 1 min bounds. Squares at 11.1 and 12.8 min correspond to proquinazid and dodemorph, respectively, which elute at high organic-phase content, represented by the solid curve.

The performance of the ML models falls within the ranges reported in the literature for retention time, ionizability, and related identificands.^36,41,43^ Therefore, these models are suitable for integration into the identification workflows presented in this study.

### Identification Performance

The performance of the identification workflow was evaluated using the extracts detailed in the Experimental Section. The total number of identified compounds and the absolute sensitivities for the SS and NTS approaches are shown in Figures 4 and 5, respectively. Algorithmic sensitivities (relative to the CRS) are summarized in Tables S4 and S6 of the Supporting Information.

**Figure 4:**
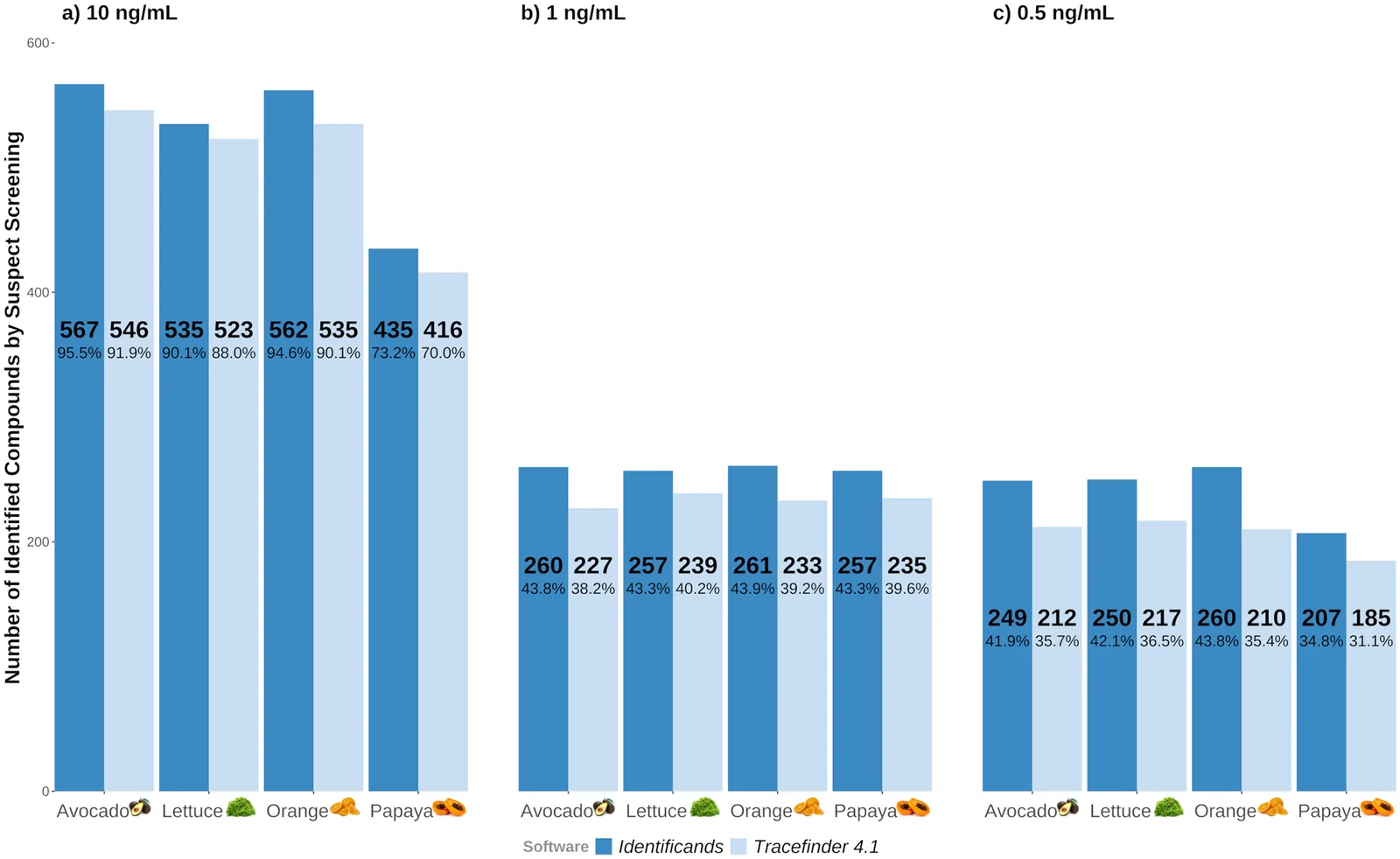
Number of compounds identified using the SS approach at three concentration levels: (a) 10 ng/mL, (b) 1 ng/mL, and (c) 0.5 ng/mL. A total of 594 compounds served as the reference set for calculating absolute sensitivity, expressed as percentages in each bar.

**Figure 5:**
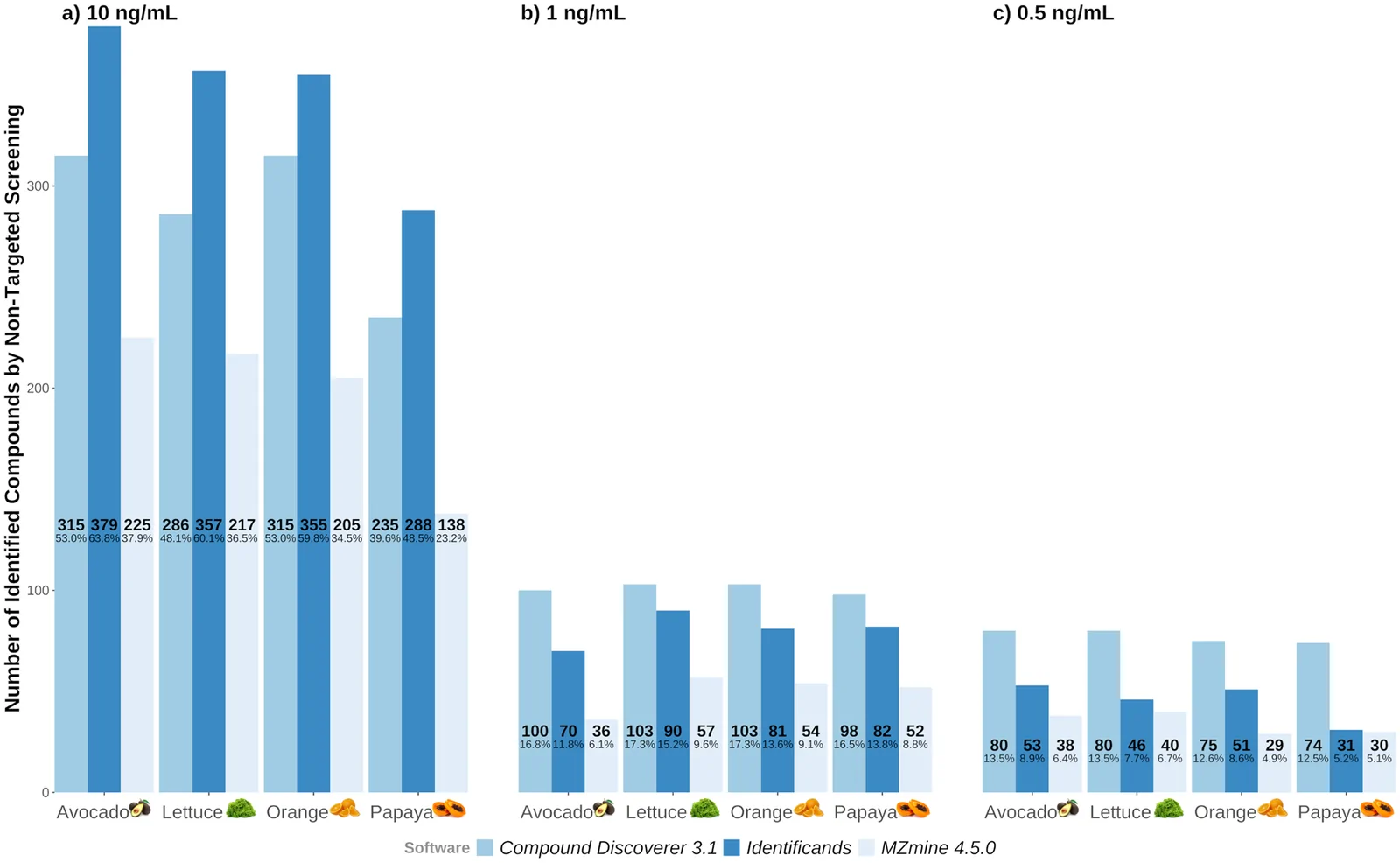
Number of compounds identified using the NTS approach at three concentration levels: (a) 10 ng/mL, (b) 1 ng/mL, and (c) 0.5 ng/mL. A total of 594 compounds served as the reference set for calculating absolute sensitivity, expressed as percentages in each bar.

In SS, *Identificands* consistently outperformed *TraceFinder* in absolute identifications (Figure 4). When evaluated against the CRS to assess the TP rate (Table S4), *Identificands* showed significantly higher sensitivity than *TraceFinder* across all concentrations, ranging from 89.1% to 97.0% between 0.5 and 10.0 ng/mL (*p* < 0.001; Table S5). This improvement results from *Identificands’* ability to dynamically extract coeluting vDIA fragments and evaluate—using the candidate’s SMILES—which ions can be attributed to that structure, often identifying more fragment ions than a user-defined inclusion list. Consequently, this reduces dependence on prior MS/MS knowledge, streamlining data analysis in routine laboratories.

For NTS, both the number of identifications and algorithmic sensitivity were concentration-dependent (Figure 5 and Table S6). At lower concentrations, *Compound Discoverer* yielded significantly higher algorithmic sensitivity than *Identificands* (28.5% vs 16.7% at 0.5 ng/mL, and 35.8% vs 28.4% at 1.0 ng/mL; *p* < 0.001; Tables S6 and S7). However, at 10.0 ng/mL, *Identificands* surpassed both *Compound Discoverer* and *MZmine*, identifying 6.7–11.8% and 23.4–25.9% more compounds, respectively. Similarly, *Identificands* achieved a significantly higher sensitivity of 63.7% relative to the CRS (*p* < 0.001; Table S7). Furthermore, the number of FPs in blank extracts was significantly lower for *Identificands* (mean = 2.7; *p* < 0.001; Tables S6 and S8). Regarding the annotation burden, *Identificands* generated significantly fewer annotations across all concentrations (≤1081; *p* < 0.001; Tables S9 and S10) compared to *Compound Discoverer* (≤2409) and *MZmine* (≤4281).

These results reflect the performance of *Compound Discoverer* and *MZmine* under default settings, and *Identificands* under developmental parameters, with only key criteria harmonized (Tables S1–S2). Consequently, comprehensive expert optimization could alter these outcomes and substantially improve the performance of any evaluated software.

### Suspect Screening vs Nontargeted Algorithm

Figures 4 and 5 show the compound identifications confirmed by retention time (within ± 0.15 min) and by the presence of characteristic fragment ions. In the SS approach, each *Identificands* search was guided by an inclusion list containing minimal compound information. The reduction in identifications relative to the 594 RCs is therefore primarily explained by matrix effects and analyte concentration. In contrast, the lower number of identifications obtained in the NTS approach reflects not only these two factors but also the cumulative performance of each step in the NTS pipeline. This reduction is inherent to the greater complexity of NTS workflows, where the search space remains unconstrained by predefined inclusion lists.

The decline in identification rate in the NTS workflow was evaluated relative to the identifiable compounds defined in the CRS. Table 3 summarizes the relative recall across the signal detection and scoring steps (identificand comparison and confidence calculation) within the *Identificands* workflow (Steps 1–3 in Figure 1). The results indicate that signal detection is the most sensitive step to concentration, with relative recall decreasing to 29% at 0.5 ng/mL. This behavior is expected, as isotopic signals are lost at low concentrations and the implemented NTS signal detection algorithm requires at least two isotopic signals to ensure confidence. In contrast, the scoring steps were less affected by concentration. On average, 84% (SD = 7%) of compounds were retained during the identificand comparison step, with losses mainly attributed to incorrect estimations such as fragment ion searches at nonoptimal retention times. The final confidence calculation and ranking step yielded an average relative recall of 73% (SD = 8%), with losses primarily associated with candidates not ranked first or with isomeric compounds exhibiting comparable confidence scores.

**Table 3:** Relative Recall (%) across the *Identificands* NTS Workflow (Analytical Signal Detection Relative to the CRS; Subsequent Stages Relative to the Preceding Stage)

Matrix	Concentration/ng mL^–1^	Number of CRS-Defined Identifiable Compounds	Relative Recall (%) in Analytical Signal Detection	Relative Recall (%) in Identificand Comparisons	Relative Recall (%) in Identification Confidence and Ranking
Avocado	0.5	276	35.1	75.3	72.6
Lettuce		279	35.1	79.6	59.0
Orange		286	33.2	82.1	65.4
Papaya		243	29.2	71.8	60.8
Avocado	1	287	46.0	78.0	68.0
Lettuce		285	51.9	83.8	72.6
Orange		287	46.7	82.8	73.0
Papaya		278	50.7	82.3	70.7
Avocado	10	583	83.2	92.6	84.4
Lettuce		535	86.0	92.4	81.7
Orange		578	81.1	92.3	82.0
Papaya		453	85.2	91.7	81.4

Overall, these findings indicate that the main bottleneck in the NTS workflow lies in signal detection, whereas scoring stages maintain more stable performance across concentration levels. Quantifying losses at each stage provides a practical means to identify where and why misidentifications occur. For analytical practitioners, this understanding supports more informed interpretation and clarifies the current limitations of the NT implementation within this framework.

### Blind Assay

A blind assay was conducted to evaluate the overall performance of the proposed workflow across the complete analytical process. The analyzed samples corresponded to real or fortified matrix extracts containing analyte mass fractions representative of those typically found in food commodities. Table 4 summarizes the results reported independently by each analyst. Although additional analytes were detected by both SS and NTS approaches, these findings are not discussed here, as the present evaluation focuses on comparison with the confirmed set of compounds.

**Table 4:** Identification Results from a Blind Assay of Five Real Samples Previously Quantified by an Independent Analyst Using HRMS-Orbitrap PRM^c^

		Quantitative Assay	Identification Assay	
Sample ID (Matrix)	Reported Compound	Mass Fraction/mg kg^–1^	SS Identification Confidence	NTA Identification Confidence
Sample 1 (Pineapple)	Thiabendazole	<0.010	0.591^a^	-
Sample 2 (Pineapple)	Thiabendazole^b^	0.021	0.741	0.744
Sample 3 (Chia Seeds)	Chlorantraniliprole	<0.010	-	-
Sample 4 (Papaya)	Azoxystrobin	<0.010	0.743	-
	Difenoconazole	0.015	0.652	-
	Imidacloprid^b^	0.050	0.751	0.728
	Imidaclorprid-olefin	0.015	0.557^a^	-
	Prochloraz^b^	0.398	0.742	0.748
	Propiconazole	0.014	0.632	-
	Tebuconazole^b^	0.024	0.722	0.729
	Trifloxystrobin^b^	0.010	0.742	-
Sample 5 (Tomato)	Acephate	0.038	0.690	-
	Bifenthrin	<0.010	-	-
	Boscalid	<0.010	0.689	-
	Clothianidin	0.012	0.701	-
	Dinotefuran	<0.010	0.771	0.444^a^
	Fipronil	<0.010	-	-
	Fluopicolide	<0.010	0.761	-
	Lambda-Cyhalothrin	<0.010	-	-
	Methamidophos	0.020	0.615	-
	Sulfoxaflor	<0.010	0.676	-
	Thiamethoxam^b^	0.016	0.749	-

a False negative discarded at the scoring stage because the observed fragment signal was not annotated as a probable fragment.

b Compounds identifiable in the NTS workflow at the sample mass fraction.

c Values <0.010 mg/kg indicate analytes below the quantification limit (0.010 mg/kg), and hyphens denote compounds not identified.

Table 4 shows SS identification rates of 87.5% for sample 4 and 72.7% for sample 5. Thiabendazole (sample 1) and imidacloprid-olefin (sample 4) were not reported because the algorithm discarded fragmentation signals not supported by *MetFrag* annotation. Furthermore, no workflow detected chlorantraniliprole (sample 3) or bifenthrin, fipronil, and lambda-cyhalothrin (sample 5). This was due to their low mass fractions and the lower sensitivity of FS-vDIA relative to the FS-PRM quantification method. Additionally, the missing compounds in sample 5 are observed as [M + NH_4_]^+^ adducts—with fipronil also exhibiting poor sensitivity.^58^ Because the identification analysis was restricted to protonated species, their absence was expected. In a targeted postanalysis using the vDIA data, no signals were detected by QuEChERS-UHPLC-HESI-HRMS-ORBITRAP FS-vDIA, confirming the lower sensitivity of this acquisition mode compared with the FS-PRM quantification method.

For the NTS workflow, the performance extracts provided a reference to evaluate identification outcomes considering the current state of the algorithm, matrix composition, and mass fraction. As shown in Table S11 (Supporting Information), at least six compounds (imidacloprid, prochloraz, tebuconazole, thiabendazole, thiamethoxam, and trifloxystrobin) should have been detectable by the NTS workflow, since their analytical signals were present and their concentrations were consistent with the observed performance. Among these, imidacloprid, prochloraz, tebuconazole, and thiabendazole were correctly identified. In contrast, thiamethoxam and trifloxystrobin were missed due to distinct WT-ANN filtering issues. Trifloxystrobin was erroneously discarded because abundant background ions skewed the static WT-power threshold (mean +2 SD), filtering out its signal (8.4 × 10^5^, 0.25% of the base peak). Addressing this limitation requires implementing dynamic thresholding in future versions of *Identificands* to preserve trace-level analytes during NTS. Thiamethoxam, meanwhile, was excluded because the ANN misassigned a spurious signal to the M+1 isotopic ion, disrupting subsequent isotopic pattern processing. Although the workflow reliably identifies most expected compounds, these cases demonstrate that limitations persist in *Identificands’* NTS signal detection, particularly regarding weak or distorted isotopic patterns.

Omitting a fragment inclusion list requires evaluating all fragments within the candidate *m*/*z* window, increasing computational demand. However, this approach reduces the need for prior knowledge and manual curation of compound-specific fragment information. The broader search space may increase the likelihood of false-positive assignments arising from matrix interferences or structurally related compounds. Nevertheless, the blind-assay results indicate that the SS workflow identifies more than 72% of trace-level compounds. Although the stricter multiparameter evaluation employed in the NTS workflow results in lower recall than vendor software, it provides a conservative strategy for contaminant identification above the quantification limit.

### Identification Confidence and Identificand Effects

The identification confidence was examined for methamidophos and prochloraz in the blind assay. These compounds, present at relatively low and high mass fractions, respectively, allowed evaluation of how analyte concentration influences identification confidence. For prochloraz, results are presented in Figures 6 and 8a. In sample 4, prochloraz was identified by both SS and NTS approaches, supported by its high mass fraction and the distinctive isotopic pattern associated with three chlorine atoms. The isotopic pattern was correctly recognized by the WT-ANN algorithm, and the NTS approach yielded an identification confidence of 0.748. This value was mainly reduced by retention time inference (9.20 ± 2.93 min), which which has a wide uncertainty and therefore produces a low inference weight (eq 4), thereby reducing the probability associated with the retention time to 0.405 and lowering the overall identification confidence by 19.7% compared with a perfect match (*p* = 1). Sulfonylbiuret, found in the CRL, is an isobar of prochloraz (Δ*m*/*z* ≈ 0.22 ppm). However, its low probabilities for retention time (*p* = 0.021), isotopic pattern (*p =* 0.0, as sulfonylbiuret is nonhalogenated), and fragmentation profile (*p* = 0.548) collectively resulted in an overall identification confidence of 0.363, allowing the algorithm to correctly discard sulfonylbiuret as the most probable candidate.

**Figure 6:**
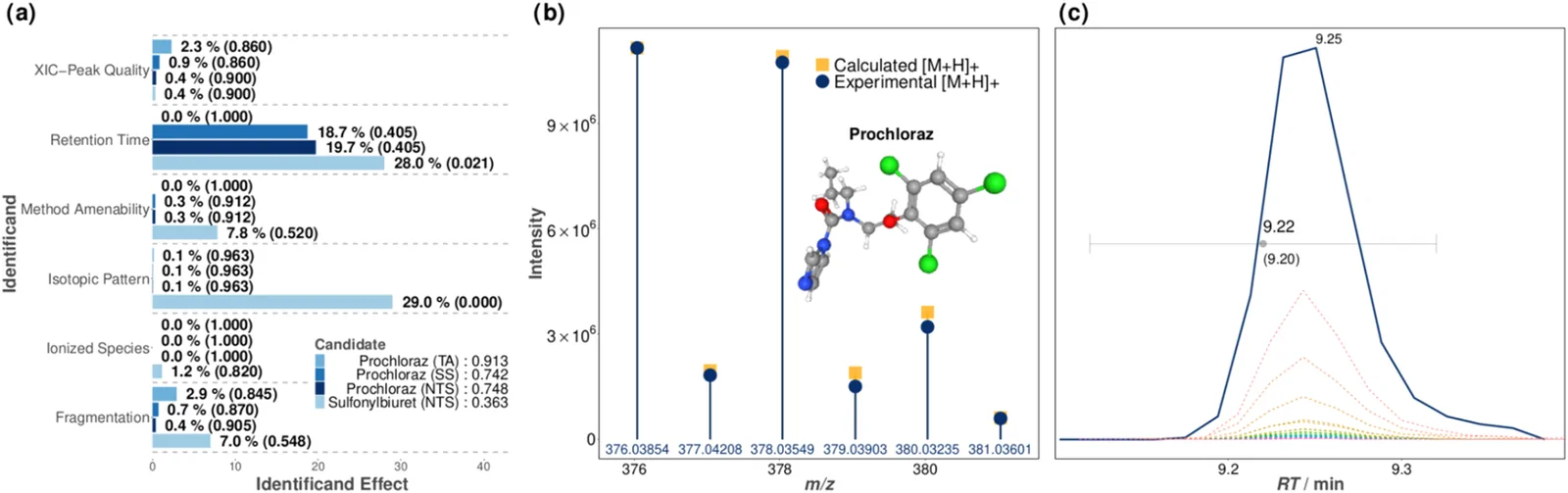
Identification results for prochloraz. (a) Identification confidence for TA, SS, and NTS, including one isobar candidate. Identificand probabilities are shown in parentheses. Bars indicate the percentage effect of each identificand to overall confidence. (b) Isotopic pattern extracted by the NTS (WT-ANN) algorithm. (c) Overlays of FS (solid) and vDIA (dashed) XICs, with the expected retention-time interval and ML-inferred value shown in parentheses.

The TA identification yielded the highest confidence value (0.913). The most influential factor was the fragmentation profile, where three of the five target ions (*m*/*z* 194.9168, 265.9538, 308.0007, Figure 8a.1) were detected with strong agreement to the FS-XIC of the protonated ion (*p* > 0.85). For the SS and NTS approaches, higher fragmentation probabilities resulted from the combined contribution of XIC similarity and *MetFrag* annotation (eqs 5–7), which identified additional probable fragment ions beyond those included in the TA inclusion list (Figures 6c and 8a.2).

Methamidophos in sample 5 at a mass fraction of 0.020 mg/kg, was identified by the SS and TA approaches. At this concentration, the NTS workflow failed to identify the compound (Section Blind Assay). Figure 7 summarizes the results. For SS, the identification confidence was 0.615, primarily limited by retention time inference and fragmentation profile (Figure 8b). The inferred retention time (4.09 min ± 1.12 min; methamidophos was not included in the training set for the retention time ML-model) deviated by 1.69 min from the expected value (2.40 min ± 0.1 min), and only one product ion (*m*/*z* 112.016 13, Figure 8b.2) was detected and annotated, resulting in a low fragmentation probability (*p* = 0.492). Regarding FS-XIC peak quality (Figure 7c), the chromatographic peak exhibited poorer morphology than that of prochloraz due to the lower analyte concentration. Nevertheless, the peak was classified as acceptable and showed high similarity with the XIC of the product ion (*p* = 0.983). These results indicate that the ML model for the XIC quality effectively evaluates peak acceptability for identification purposes (*p* = 0.682). This information is also incorporated into the TA approach, which achieved an identification confidence of 0.755. Other identificands had minimal influence on the overall confidence. However, the isotopic pattern showed a high probability (*p* = 0.991), as two isotopic signals were detected despite the low analyte concentration, contributing positively to the final confidence score.

**Figure 7:**
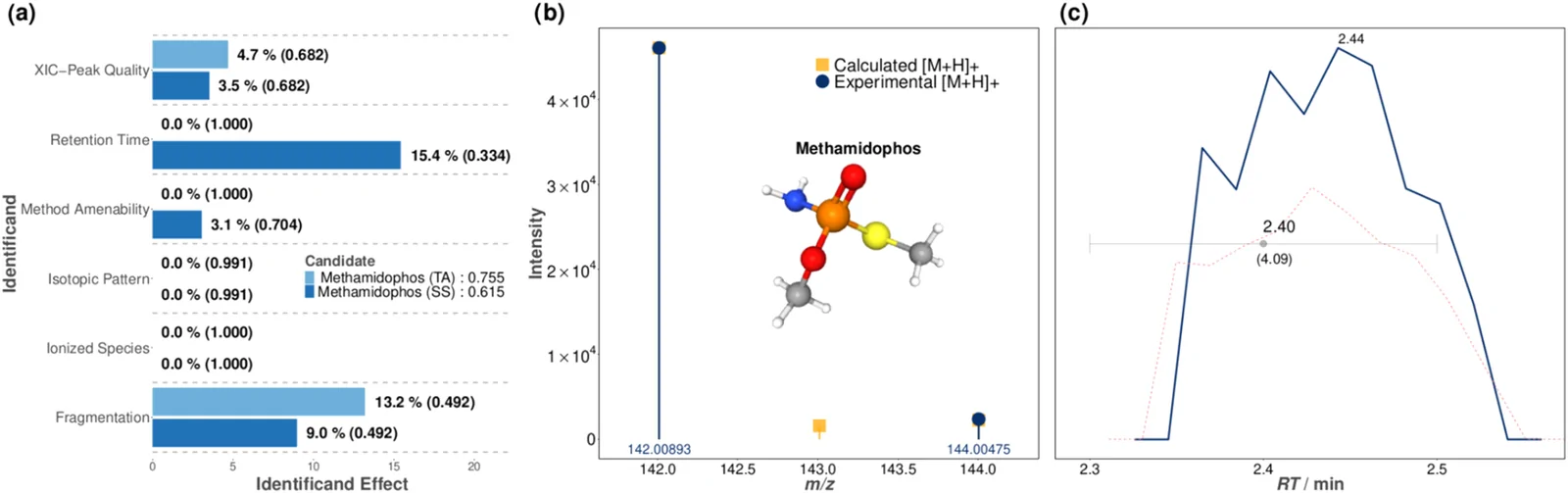
Identification results of methamidophos. (a) Identification confidence for TA, SS, and NTS. Identificand probabilities are shown in parentheses. Bars indicate the percentage effect of each identificand on overall confidence. (b) Isotopic pattern detected by the SS approach. (c) Overlays of FS (solid) and vDIA (dashed) XICs, with the expected retention-time interval and ML-inferred value shown in parentheses.

**Figure 8:**
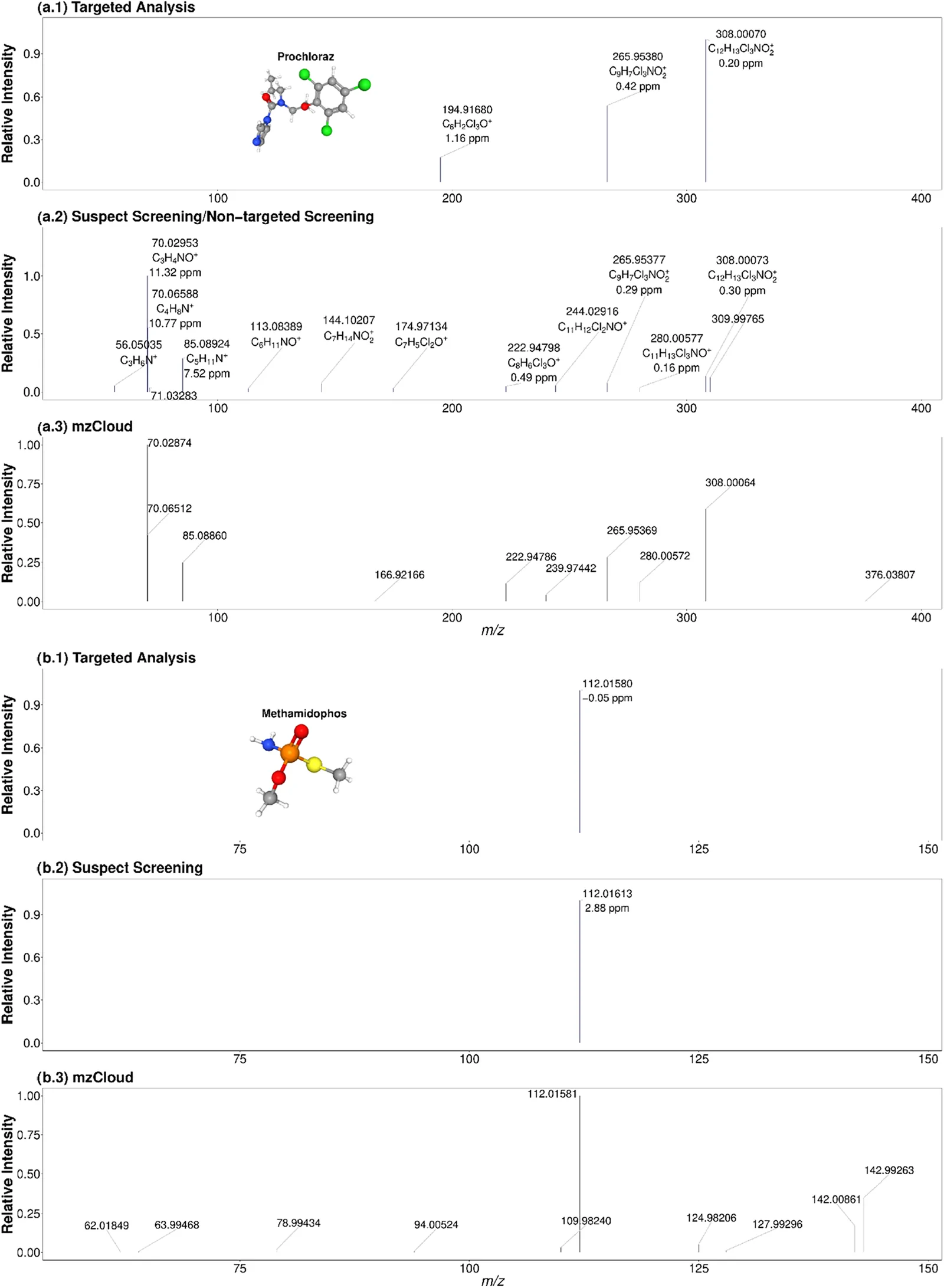
MS/MS fragmentation spectra of (a) prochloraz and (b) methamidophos obtained using the TA, SS, and NTA identification approaches. Experimental reference spectra from mzCloud (CE = 30 eV) are included for comparison. For peaks matching the reference, mass errors (in ppm) were calculated relative to their theoretical *m*/*z* values.

The results for prochloraz and methamidophos indicated high probabilities for amenability and ionized species identificands, suggesting that these factors contributed minimally to the overall identification confidence. Because this study focused exclusively on protonated species, and only one additional protonated isobar of prochloraz is present in the CRL, amenability and ionized species identificands can be regarded as negligible under these conditions. In contrast, within the NTS approach (where multiple candidates such as isobars or isomers coexist within the chemical space) these identificands become more informative and significantly influence the identification outcome.

The proposed framework aligns conceptually with the Schymanski Level 1 (confirmed structure) for TA, and Level 2 (probable structure) and Level 3 (tentative candidates) for SS and NTS, respectively.^19,73^ Identification criteria in pesticide regulatory guidelines (e.g., SANTE,^74^ CODEX,^75^ US FDA^76^) correspond to Schymanski Level 1 for target analysis and target screening, where compounds are known in advance. However, these regulatory guidelines are restricted to this level, whereas the Schymanski scale provides a basis for exploratory tasks (Levels 2 and 3 for SS and NTS) that fall outside the scope of current formal validation in pesticide laboratories. Future work could explore probabilistic ML approaches to calibrate proposed identification scores to these discrete confidence boundaries.

### Amenability and Ionized Species

Table S12 (Supporting Information) shows examples of compounds identified by the NTS approach in the avocado performance extract at 10 ng/mL, where methodological amenability and ionized species identificands were particularly relevant. Oxasulfuron and diclocymet exemplify highly ionizable targets that are correctly prioritized over poorly ionizing candidates when evaluated using the ionized species and amenability identificands. For example, two isobars for oxasulfuron were present in the CRL. The isobaric candidate 1-thio-β-d-glucose pentaacetate exhibited low probabilities for the protonated species (*p* = 0.520), retention time (*p* = 0.438; < 0.5 due to the inference step in the NTS approach, eq 4), methodological amenability (*p* = 0.416), and fragmentation profile (*p* = 0.130), while other identificands exceeded 0.669. Between the methodological amenability and fragmentation profile, the latter contributed more to discrimination; however, the low probabilities of the protonated species and methodological amenability further supported the rejection of this false candidate, lowering its overall identification confidence. A similar pattern was observed for diclocymet, which had three isobars in the CRL.

Since ESI favors protonated and deprotonated species, the ionized species identificand is useful for inferring alternative adducts when the protonated form is absent. For instance, lambda-cyhalothrin’s low probabilities (*p*_protonated_ = 0.220; *p*_deprotonated_ = 0.120) correctly suggest other adducts, aligning with its reported [M + NH_4_]^+^ preference.^58^ However, the ML model’s protonation bias (e.g., bifenthrin, *p*_protonated_ = 0.885) requires caution, as it could drive incorrect annotations from false-positive signals. Ethoprophos and edifenphos (Table S12) further exemplify cases where methodological amenability and ionized species identificands were essential in reducing the number of candidates, thereby increasing the confidence in correct compound identification.

In Table S12, tebutam represents a case where the ionized species probability is high (≥0.95), while methodological amenability for the other candidates is low (<0.60). The tebutam group comprises nine candidates within the CRL. Seven of the eight alternative candidates exhibit further reduced confidence due to low inferred probabilities for methodological amenability. While fragmentation profile probabilities were below 0.50 for four candidates, methodological amenability was decisive for excluding the remaining four alternatives in favor of tebutam. These findings highlight the complementary role of the methodological amenability and ionized species identificands in discriminating among probable candidates, thereby improving the robustness of NTS-based identification.

The results also establish a framework for interpreting how identification outcomes are reached and for identifying which identificands contribute most to uncertainty. This framework is valuable not only for understanding identification performance but also for comparing data-analysis methods and identifying opportunities to improve inference algorithms or experimental protocols. Although demonstrated here using six identificands, the approach can be extended to additional identification properties and other analytical techniques, thereby enhancing confidence in both targeted and nontargeted identification. By improving interpretability, benchmarking, and cross-method comparability, this framework provides a practical foundation for advancing data-driven identification strategies in analytical chemistry.

## Conclusions

An ML-assisted software workflow was developed to support SS and NTS analysis using a QuEChERS-HPLC-ESI-HRMS Orbitrap method for pesticide residue analysis in fruits and vegetables. The workflow integrates multiple identification properties inherent to the analytical methodology into a unified scoring scheme. Both the conceptual framework and its software implementation demonstrated improved performance compared with some existing tools at high concentrations (≥10 ng/mL), where the proposed approach identified more than 6% additional compounds. A key strength of the workflow lies in its flexibility. The SS approach requires minimal input information to guide identification, whereas the NTS workflow leverages all available analytical and experimental data incorporated into ML models. Methodological amenability is modeled through ML, while the use of vDIA MS/MS information enhances identification in both SS and NTS settings. By unifying quantitative and qualitative identification properties, termed identificands, the workflow increases identification confidence and produces results that are directly interpretable. Although the approach involves assumptions regarding identificand probabilities, relies on a simple combination model, and exhibits limitations in NTS signal detection step, the evaluation at low mass fractions demonstrated its capability to identify pesticide residues under both SS and NTS analysis.

The analysis of identificand effects, conceptually analogous to uncertainty budgets in quantitative analysis, improves the interpretability and reliability of chemical identification. By clarifying the contribution of each identificand to the final result, the proposed framework provides transparent and reproducible decision criteria suitable for routine laboratories, where defensible conclusions are essential. Moreover, it enables the integration and cross-comparison of TA, SS, and NTS identification strategies within a consistent framework.

In summary, *Identificands* yields high algorithmic sensitivity in SS and strictly controls annotation burden in NTS. By pairing vDIA MS/MS data filtering with library-free structural annotation, it functions as a rapid prospective screening tool that streamlines routine workflows, flagging tentative candidates for targeted confirmation to continuously expand regulatory-compliant methods (e.g., SANTE, CODEX, FDA). However, NTS sensitivity decreases at trace levels (5 μg/kg and 10 μg/kg), where *Compound Discoverer* detected significantly more compounds. This loss of analytical signals at low mass fractions underscores the need for improved detection algorithms. Future efforts should focus on expanding the ML training data sets, evaluating compounds not represented in model development, exploring additional chemical spaces, and extending the MS/MS fragmentation identificand to process DDA data. Expanded training data sets will also enable the incorporation of additional ionized species beyond protonated and deprotonated molecules.. Furthermore, the framework remains extensible; additional identificands can be integrated, and the identification confidence metric could serve as a prior probability for incorporating complementary evidence.

## Supplementary Material



## Data Availability

The *Identificands* source code, machine learning data sets, and training scripts are publicly available on GitHub (https://github.com/sagm-co/msLabAIssistant). Benchmarking data sets are archived on Zenodo (10.5281/zenodo.19135094).

## Notes

Identificand parameters and thresholds (Table S1); harmonized workflow parameters and identification resources for comparative software evaluation (Table S2); individual high-purity standards used for the preparation of Working Solution B at 10 μg/mL (Table S3); *Identificand* performance metrics for SS and NTS workflows (Tables S4–S10); comparison of expected and observed identification outcomes for blind assay analytes (Table S11); NTS identification confidence and identificand probabilities from *Identificands* for selected candidate analytes in avocado extract at 10 ng/mL (Table S12); and a glossary of acronyms and key technical terms (PDF)
