# Supplementary material for: Machine Learning–Enhanced LC–HRMS Workflows for Suspect and Nontargeted Screening of Pesticide Residues in Fruits and Vegetables
Source: Anal Chem. 2026 Aug 24;98(35):25557–74. doi: 10.1021/acs.analchem.5c06726 (PMC13573562; doi:10.1021/acs.analchem.5c06726)
Supplement: Supplementary file 1 [file ac5c06726_si_001.pdf]

Supporting Information to: Machine Learning–Enhanced LC–HRMS Workflows for Suspect and Non–Targeted Screening of Pesticide Residues in Fruits and Vegetables

Sergio González,† Gabriel Merino,*,‡ and Jairo Arturo Guerrero Dallos*,†

†*Departamento de Química, Universidad Nacional de Colombia, 111321, Bogotá D.C., Colombia*

‡*Departamento de Física Aplicada, Centro de Investigación y de Estudios Avanzados, 97310 Mérida, México*

E-mail: gmerino@cinvestav.mx; jaguerrerod@unal.edu.co

This Supporting Information document (SI) consists of 25 pages and contains 12 tables.

Contents

S1 Workflow Performance Evaluation **S3**

S1.1 Statistical Modeling of Detection Sensitivity and the Algorithmic False Negative Rate . . . S4

S1.2 Statistical Modeling of False Positive Counts in Blank Matrix Extracts . . . S5

S1.3 Statistical Modeling of Software Annotation Burden in Non-Targeted Screening S6

S2 Tables **S7**

S3 Glossary of Acronyms and Key Technical Terms **S15**

S3.1 Machine Learning & Data Science S15

S3.2 Cheminformatics Tools S18

S3.3 Metrological & Analytical Concepts S18

S3.4 Acronyms & Abbreviations S20

References **S23**

List of Tables

S1 *Identificands* parameters and thresholds S7

S2 Harmonized workflow parameters and identification resources for comparative software evaluation. m/z tolerances, intensity thresholds, and identification resources were aligned across platforms; all other settings remained at software-specific defaults. S8

S3 Individual high-purity standards used for preparation of working solution B at 10 μg/mL.. . . . . S9

S4 Performance metrics for suspect screening (SS) software across four food matrices (avocado, lettuce, orange, and papaya). Data are derived from generalized linear models (GLM) using binomial and COM-Poisson families. Matrix type was incorporated as a blocking factor to account for matrix-specific effects. S9

S5 Pairwise comparisons of sensitivity (TPR) between software packages across concentration levels for the suspect screening (SS) workflow S10

S6 Performance metrics for non-targeted screening (NTS) software across four food matrices (avocado, lettuce, orange, and papaya). Data are derived from generalized linear models (GLM) using quasibinomial and COM-Poisson families. Matrix type was incorporated as a blocking factor to account for matrix-specific effects. S10
S7 Pairwise comparisons of sensitivity (TPR) between software packages across concentration levels for the non-targeted screening (NTS) workflow S10
S8 Pairwise comparisons between software packages for false positive counts in blank matrix extracts (NTS workflow) S11
S9 Annotation burden for non-targeted screening (NTS) software across four food matrices (avocado, lettuce, orange, and papaya). Data are derived from generalized linear models (GLM) using negative binomial distribution. Matrix type was incorporated as a blocking factor to account for matrix-specific effects S11
S10 Pairwise comparisons of annotation burden between software packages across concentration levels for the non-targeted screening (NTS) workflow S12
S11 Comparison of expected and observed identification outcomes for blind assay analytes based on performance assessment results. S13
S12 NTS identification confidence and identificand probabilities from *Identificands* for selected candidate analytes in avocado extract (10 ng/mL). The candidate groups shown are examples where methodological amenability and the ionized species (protonated species) are relevant. S14

S1 Workflow Performance Evaluation

Performance metrics for suspect screening (SS) reflect the workflow's ability to selectively extract and identify target compounds when only partial prior information (e.g., exact masses or molecular formulas) is available. In contrast, non-targeted screening (NTS) metrics

evaluate the ability of the software to blindly detect and annotate spiked compounds treated as unknowns.

Although the evaluated platforms support full NTS workflows, comprehensive validation requires a fully characterized metabolome or exposome. Because uncharacterized matrix features cannot be exhaustively confirmed under the current experimental design, the evaluation of absolute identification performance falls outside the scope of this study. Consequently, the calculation of absolute sensitivity and global false-positive (FP) rates was not feasible.

To provide a verifiable benchmark, the evaluation was restricted to the ability of each workflow to correctly identify compounds within a consensus reference set (CRS) treated as unknowns (DOI: 10.5281/zenodo.19135094, file: `Consensus_Reference_Set_CRS.tsv`). This approach isolates algorithmic performance from generalized feature-annotation capabilities. To assess the comparative workload, the total number of annotated candidate features—independent of confident confirmation—was included as a secondary metric (annotation burden).

Reported SS and NTS FP counts are limited to false identifications of the validation compounds in blank matrix extracts. This metric reflects algorithmic specificity for the target compounds and does not represent the global FP rate across the full untargeted dataset.

Identification results for each software package and the corresponding R[1] analysis script are available on Zenodo (DOI: 10.5281/zenodo.19135094, directory: `performance_evaluation`)

S1.1 Statistical Modeling of Detection Sensitivity and the Algorithmic False Negative Rate

To evaluate sensitivity, the True Positive Rate (TPR) was modeled independently for the SS and NTS workflows. For each experimental unit (software i at concentration j in matrix k), the number of correctly identified compounds (TP_{ijk}) relative to the total compounds in the CRS (N_{jk}) was treated as a binomial random variable:

$$TP_{ijk} \sim \text{Binomial}(N_{jk}, p_{ijk}). \tag{S1}$$

A generalized linear model (GLM) with a logit link function was employed to estimate the detection probability (p_{ijk}):

$$\text{logit}(p_{ijk}) = \beta_0 + S_i + C_j + (SC)_{ij} + M_k, \tag{S2}$$

where β_0 is the intercept, S_i is the software effect, C_j is the concentration effect, and $(SC)_{ij}$ is their interaction. The matrix (M_k) was incorporated as an additive blocking factor to account for matrix-specific effects. Post-hoc evaluation was conducted using the `emmeans` package[2] in R[1] to compute estimated marginal means (EMMs),which are reported on the probability scale with 95% confidence intervals. The algorithmic false negative rate (FNR) was subsequently calculated as $1 - p_{ijk}$ (Tables S4 and S6). Model assumptions, were verified using simulated residual analysis via the `DHARMa` package[3]. Pairwise contrasts between software platforms were calculated using the `pairs` function in R (Table S5 and S7).

S1.2 Statistical Modeling of False Positive Counts in Blank Matrix Extracts

The number of False Positives (FP) detected in unspiked (blank) extracts was modeled independently for the SS and NTS workflows. For each experimental unit (software i in matrix k), the false positive count (FP_{ik}) was modeled using a Conway-Maxwell-Poisson (COM-Poisson) distribution[4], because diagnostic testing revealed underdispersion for both workflows (simulated dispersion ratios of 0.35 and 0.16, respectively):

$$FP_{ik} \sim \text{COM-Poisson}(\lambda_{ik}, \nu). \tag{S3}$$

A GLM with a log link function was fitted to estimate the expected false positive count

(λ_{ik}):

$$\log(\lambda_{ik}) = \beta_0 + S_i + M_k, \tag{S4}$$

where β_0 is the intercept, S_i is the software effect, and M_k is an additive blocking factor to account for matrix-specific effects. Post-hoc evaluation of the software effect was conducted using the `emmeans` package in R to compute estimated marginal means (EMMs), which are reported on the response (count) scale with 95% confidence intervals (Tables S4 and S6). Model assumptions, including the absence of zero-inflation, were verified using simulated residual analysis via the `DHARMa` package. Pairwise contrasts between software platforms were calculated using the `pairs` function in R (Table S8).

S1.3 Statistical Modeling of Software Annotation Burden in Non-Targeted Screening

The annotation burden (total annotated compounds requiring review or confirmation for complete identification) was modeled using a Negative Binomial (NB) generalized linear model (GLM) with a log link function. Initial diagnostic testing of a standard Poisson model revealed significant overdispersion (simulated dispersion ratio = 3.68, $p < 0.001$). To appropriately account for this, the NB1 parameterization was employed, which assumes the variance scales linearly with the mean. This approach accounted for the excess variance, reducing the simulated dispersion ratio to 0.98 ($p = 0.96$) in the final NB1 model. The model is defined as:

$$\ln(\lambda_{ijk}) = \beta_0 + S_i + C_j + (SC)_{ij} + M_k, \tag{S5}$$

where λ_{ijk} is the expected annotation count, β_0 is the intercept, S_i is the software effect, C_j is the concentration effect, and $(SC)_{ij}$ is their interaction. The matrix (M_k) was incorporated as an additive blocking factor to account for matrix-specific effects. Model assumptions were verified using simulated residuals via the `DHARMa` package. Estimated Marginal Means

(EMMs) and 95% confidence intervals were computed to evaluate software performance across concentration levels (Table S9). Finally, pairwise contrasts between software platforms were calculated using the `pairs` function in R (Table S10).

S2 Tables

Table S1: *Identificands* parameters and thresholds

Equation		Parameter	Value	Unit	Description
$e_v = \frac{\mathbf{v}_{\mathcal{A}}\cdot\mathbf{v}_{\mathcal{R}} - \mathbf{v}_{\mathcal{R}}\cdot\mathbf{v}_{\mathcal{R}}}{\mathbf{d}_v\cdot\mathbf{v}_{\mathcal{R}}}$,	equation 2 in the main text	$\mathbf{d}_a$	$[0.01; 0.35]$	–	Intensity deviation vector. Thresholds of 10% for the monoisotopic signal and 35% for non-monoisotopic signals.
		$\mathbf{d}_m$	$[5.0; 5.0]$	ppm	m/z deviation vector. A tolerance of 5 ppm is applied to both monoisotopic and non-monoisotopic signals.
$p(\text{match}_s \mid \mathcal{R}) = \frac{P\{S \leq s \mid \lvert S-\mu_S\rvert \leq k\sigma_S, \lvert S\rvert \leq c_S\}}{P\{\lvert S-\mu_S\rvert \leq k\sigma_S\}}$,	equation 3 in the main text	k	1.96	–	95% confidence level assuming a normal distribution.
		c_s	0.15	min	Retention-time acceptance criterion for targeted analysis.
		c_s	2RMSE	min	Retention-time acceptance criterion for SS and NTS based on machine learning (ML)-predicted retention times. RMSE, root-mean-square error.
		σ_s	0.01 or $s_{\text{RT,pred}}$	min	0.01 min for experimental measurements and $s_{\text{RT,pred}}$ from the ML-based retention-time prediction model.
		c_s	$\sqrt{2}$	–	Acceptance criterion for the isotopic-pattern error.
		σ_s	$\sqrt{2}/k$	–	Assumed standard deviation of the isotopic-pattern deviation.
$w_{\text{RT}} = \frac{1}{1+\log_{10}\max\left(1, U_{\text{RT,ref}}/U_{\text{RT,measured}}\right)}$,	equation 4 in the main text	$U_{\text{RT,measured}}$	0.1	min	Assumed uncertainty of the measured retention time.
		$U_{\text{RT,ref}}$	0.1 or $U_{\text{RT,pred}}$	min	0.1 min for measured retention times of reference materials; $U_{\text{RT,pred}}$ estimated from the ML-based retention-time prediction model.
$s_{\text{FS-MS2},i} = \frac{\lVert\mathbf{I}_{\text{FS}}-\mathbf{I}_{\text{MS2},i}\rVert^2}{n\sigma_d^2}$,	equation 5 in the main text	σ_d	0.2	–	Intensity deviation threshold

Table S2: Harmonized workflow parameters and identification resources for comparative software evaluation. m/z tolerances, intensity thresholds, and identification resources were aligned across platforms; all other settings remained at software-specific defaults.

	Tracefinder	*Identificands*	*Compound Discoverer*	*MZmine*
Software version	4.1	–	3.1	4.5.0
Licensing	propietary	open-source	propietary	open-source
Raw input format	.raw	.raw	.raw	.mzML[a]
Targeted species	$[M+H]^+$	$[M+H]^+$	$[M+H]^+$	$[M+H]^+$
Reference database[b]	Suspect list	Suspect list for SS and Compiled Reference Library (CRL) for NTS	Compiled Reference Library (CRL) used as a mass list	Compiled Reference Library (CRL) used as a mass list
Spectral libraries	–	–	mzCloud[?], ChemSpider[?]	MoNa - Mass Bank of North America (All Experimental Spectra)[5]
Customized workflow	–	–	Untargeted environmental research ID workflow without statistics	Custom Batch Pipeline
m/z tolerance (ppm)	5	5	5	5
Minimum number of fragment ions[c]	1	1	–	–
Suspect screening parameters	Isotopic score $\geq 60\%$, Minimum number of isotopic peaks: 1, Intensity threshold: 2×10^4	Minimum number of isotopic peaks: 1, intensity threshold: 2×10^4	–	–
Non-targeted screening parameters[d]	–	Minimum number of isotopic peaks: 2, intensity threshold: 5×10^4	Minimum number of isotopic peaks: 2, intensity threshold: 5×10^4	Intensity threshold: 5×10^4
Retention time tolerance (min)[e]	0.15	0.15	0.15	0.15

[a] Converted from .raw files using ProteoWizard.[6]

[b] Database files on Zenodo (DOI: 10.5281/zenodo.19135094, directory: `reference_db`).

[c] No minimum number of fragment ions was specified for *Compound Discoverer* or *MZmine*.

[d] No minimum number of isotopic peaks was specified for *MZmine*. For *Compound Discoverer* and *Identificands*, a minimum of two isotopic signals was required to ensure identification confidence.

[e] For SS, retention times were not input into the software. For both SS and NTS, retention time tolerances were evaluated during post-processing of the generated results.

Table S3: Individual high-purity standards used for preparation of working solution B at 10 μg/mL..

Compound Name	Provider	Brand	Stock Solution Solvent
Albendazole	Merck	Sigma Aldrich, Saint Louis,MO, USA	Acetonitrile/Methanol (50:50, v/v)
Albendazole sulfone	Merck	Sigma Aldrich, Saint Louis,MO, USA	Acetonitrile/Methanol (50:50, v/v)
Albendazole sulfoxide	Merck	Sigma Aldrich, Saint Louis,MO, USA	Methanol
Amoxicillin	Merck	Sigma Aldrich, Saint Louis,MO, USA	Acetonitrile/Methanol (50:50, v/v)
Ampicillin	LGC	Dr. Ehrenstorfer, Augsburg, Germany	Methanol
Benzylpenicillin	LGC	EDQM, Strasbourg, France	Methanol
Ceftiofur	Merck	Sigma Aldrich, Saint Louis,MO, USA	Acetonitrile/Methanol (50:50, v/v)
Cephalexin	Merck	USP, Hyderabad, India	Methanol
Chloramphenicol	Merck	Sigma Aldrich, Saint Louis,MO, USA	Methanol
Chlortetracycline	Merck	Sigma Aldrich, Saint Louis,MO, USA	Methanol
Danofloxacin	Merck	Sigma Aldrich, Saint Louis,MO, USA	Acetonitrile
Enrofloxacin	Merck	Sigma Aldrich, Seelze, Germany	Acetonitrile
Eprinomectin B1a	Merck	Sigma Aldrich, Saint Louis,MO, USA	Acetonitrile
Florfenicol	LGC	Dr. Ehrenstorfer, Augsburg, Germany	Acetonitrile
Flubendazole	LGC	EDQM, Strasbourg, France	Acetonitrile/Methanol/Dimethyl sulfoxide (50:49:1, v/v/v)
Gentamicin	LGC	Sigma Aldrich, Seelze, Germany	Water
Lincomycin	Merck	Sigma Aldrich, Saint Louis,MO, USA	Methanol
Mebendazole	Merck	Sigma Aldrich, Saint Louis,MO, USA	Acetonitrile/Methanol/Dimethyl sulfoxide (50:49:1, v/v/v)
Neo Spiramycin I	LGC	TRC, North York, ON, Canada	Acetonitrile
Neomycin	LGC	EDQM, Strasbourg, France	Water
Nicarbazine	LGC	Dr. Ehrenstorfer, Augsburg, Germany	Acetonitrile/Methanol/Dimethyl sulfoxide (50:49:1, v/v/v)
Oxytetracycline	Merck	Sigma Aldrich, Seelze, Germany	Methanol
Spiramycin	LGC	Sigma Aldrich, Saint Louis,MO, USA	Methanol
Stanozolol	LGC	EDQM, Strasbourg, France	Methanol
Streptomycin	LGC	Sigma Aldrich, Saint Louis,MO, USA	Acetonitrile/Water (50:50, v/v)
Sulfadiazine	Merck	Sigma Aldrich, Seelze, Germany	Acetonitrile/Methanol (80:20, v/v)
Sulfamethazine	Merck	Sigma Aldrich, Saint Louis,MO, USA	Methanol
Sulfapyridine	Merck	Sigma Aldrich, Saint Louis,MO, USA	Methanol
Sulfaquinoxaline	Merck	Sigma Aldrich, Saint Louis,MO, USA	Acetonitrile/Methanol (70:30, v/v)
Tetracycline	Merck	Sigma Aldrich, Saint Louis,MO, USA	Methanol
Thiamphenicol	LGC	EDQM, Strasbourg, France	Methanol
Tilmicosin	Merck	Sigma Aldrich, Saint Louis,MO, USA	Methanol
Tylosin	LGC	EDQM, Strasbourg, France	Methanol

[a] Additional data for these standards and compounds in Smart Solutions™ v700 PestiMix kit on Zenodo (DOI: 10.5281/zenodo.19135094, file `Table_S3_Analytical_Standards_Suite_778.tsv`)

Table S4: Performance metrics for suspect screening (SS) software across four food matrices (avocado, lettuce, orange, and papaya). Data are derived from generalized linear models (GLM) using binomial and COM-Poisson families. Matrix type was incorporated as a blocking factor to account for matrix-specific effects.

**Software**	**Mass concentration[a]** (ng/mL)	**SS Sensitivity (%) Mean [95% CI]**	**Algorithmic False Negative Rate (%) Mean [95% CI]**	**SS False Positives[b,c] (Count/Blank) Mean [95% CI]**
Tracefinder 4.1	0.5	76.0 [73.4–78.5]	24.0 [21.5–26.6]	31.7 [28.8–34.8]
	1	82.2 [79.8–84.3]	17.8 [15.7–20.2]	
	10	93.3 [92.2–94.3]	6.7 [5.7– 7.8]	
Identificands	0.5	89.1 [87.1–90.8]	10.9 [9.2–12.9]	23.9 [21.5–26.7]
	1	91.0 [89.2–92.6]	9.0 [7.4–10.8]	
	10	97.0 [96.2–97.6]	3.0 [2.4– 3.8]	

[a] 1 ng/mL corresponds to 0.01 mg/kg in the matrix.
[b] Aggregated results from four blank matrix extracts ($n = 4$)
[c] Rate ratio (RR) for the *Tracefinder* 4.1/*Identificands* comparison: 1.3 (95% CI: 1.2–1.5; $p < 0.001$).

Table S5: Pairwise comparisons of sensitivity (TPR) between software packages across concentration levels for the suspect screening (SS) workflow

**Mass concentration[a] (ng/mL)**	**Contrast**	**Odds Ratio (OR) [95% CI]**	*p*-value
0.5	*Tracefinder* 4.1 / *Identificands*	0.39 [0.31–0.49]	< 0.001
1	*Tracefinder* 4.1 / *Identificands*	0.45 [0.35–0.58]	< 0.001
10	*Tracefinder* 4.1 / *Identificands*	0.43 [0.32–0.59]	< 0.001

[a] 1 ng/mL corresponds to 0.01 mg/kg in the matrix.

Table S6: Performance metrics for non-targeted screening (NTS) software across four food matrices (avocado, lettuce, orange, and papaya). Data are derived from generalized linear models (GLM) using quasibinomial and COM-Poisson families. Matrix type was incorporated as a blocking factor to account for matrix-specific effects.

**Software**	**Mass concentration[a] (ng/mL)**	**NTS Sensitivity (%) Mean [95% CI]**	**Algorithmic False Negative Rate (%) Mean [95% CI]**	**SS False Positives[b] (Count/Blank) Mean [95% CI]**
Compound Discoverer 3.1	0.5	28.5 [25.9–31.2]	71.5 [68.8–74.1]	7.9 [6.7– 9.4]
	1	35.5 [32.8–38.3]	64.5 [61.7–67.2]	
	10	53.1 [51.0–55.2]	46.9 [44.8–49.0]	
Identificands	0.5	16.7 [14.6–19.0]	83.3 [81.0–85.4]	2.7 [2.0– 3.7]
	1	28.4 [25.8–31.1]	71.6 [68.9–74.2]	
	10	63.7 [61.6–65.7]	36.3 [34.3–38.4]	
MZmine 4.5.0	0.5	12.6 [10.8–14.7]	87.4 [85.3–89.2]	15.1 [13.4–17.0]
	1	17.5 [15.4–19.8]	82.5 [80.2–84.6]	
	10	36.2 [34.2–38.3]	63.8 [61.7–65.8]	

[a] 1 ng/mL corresponds to 0.01 mg/kg in the matrix.
[b] Aggregated results from four blank matrix extracts ($n = 4$)

Table S7: Pairwise comparisons of sensitivity (TPR) between software packages across concentration levels for the non-targeted screening (NTS) workflow

**Mass concentration[a] (ng/mL)**	**Contrast**	**Odds Ratio (OR) [95% CI]**	*p*-value (Tukey-adjusted)
0.5	*Identificands* / *Compound Discoverer* 3.1	0.50 [0.39–0.64]	< 0.001
	MZmine 4.5.0 / *Compound Discoverer* 3.1	0.36 [0.28–0.47]	< 0.001
	Identificands / *MZmine* 4.5.0	1.39 [1.04–1.85]	0.021
1	*Identificands* / *Compound Discoverer* 3.1	0.72 [0.58–0.89]	0.001
	MZmine 4.5.0 / *Compound Discoverer* 3.1	0.39 [0.31–0.49]	0.001
	Identificands / *MZmine* 4.5.0	1.87 [1.47–2.38]	< 0.001
10	*Identificands* / *Compound Discoverer* 3.1	1.55 [1.34–1.79]	< 0.001
	MZmine 4.5.0 / *Compound Discoverer* 3.1	0.50 [0.43–0.58]	< 0.001
	Identificands / *MZmine* 4.5.0	3.09 [2.66–3.58]	< 0.001

[a] 1 ng/mL corresponds to 0.01 mg/kg in the matrix.

Table S8: Pairwise comparisons between software packages for false positive counts in blank matrix extracts (NTS workflow)

**Contrast**	**Rate Ratio (RR) [95% CI]**	p-value (Tukey-adjusted)
Identificands / *Compound Discoverer* 3.1	0.35 [0.23–0.52]	< 0.001
MZmine 4.5.0 / *Compound Discoverer* 3.1	1.91 [1.49–2.43]	< 0.001
Identificands / *MZmine* 4.5.0	0.18 [0.12–0.27]	< 0.001

Table S9: Annotation burden for non-targeted screening (NTS) software across four food matrices (avocado, lettuce, orange, and papaya). Data are derived from generalized linear models (GLM) using negative binomial distribution. Matrix type was incorporated as a blocking factor to account for matrix-specific effects

**Software**	**Mass concentration**[a] (ng/mL)	**Annotations (Count) Mean [95% CI]**
Compound Discoverer 3.1	0	1198.6 [1135.1-1265.6]
	0.5	1652.1 [1577.3–1730.5]
	1	1777.8 [1700.1–1859.0]
	10	2409.4 [2318.7–2503.7]
Identificands	0	215.8 [189.9– 245.3]
	0.5	422.2 [385.2– 462.7]
	1	521.5 [480.2– 566.3]
	10	1081.1 [1020.9–1144.9]
MZmine 4.5.0	0	2812.6 [2714.4–2914.4]
	0.5	3183.8 [3079.2–3291.9]
	1	3239.6 [3134.0–3348.7]
	10	4281.0 [4159.4–4406.2]

[a] 1 ng/mL corresponds to 0.01 mg/kg in the matrix.

Table S10: Pairwise comparisons of annotation burden between software packages across concentration levels for the non-targeted screening (NTS) workflow

**Mass concentration[a]** (ng/mL)	**Contrast**	**Rate Ratio (RR) [95% CI]**	p-value (Tukey-adjusted)
0	*Identificands* / *Compound Discoverer* 3.1	0.18 [0.15–0.21]	< 0.001
	MZmine 4.5.0 / *Compound Discoverer* 3.1	2.35 [2.17–2.54]	< 0.001
	Identificands / *MZmine* 4.5.0	0.08 [0.07–0.09]	< 0.001
0.5	*Identificands* / *Compound Discoverer* 3.1	0.26 [0.23–0.29]	< 0.001
	MZmine 4.5.0 / *Compound Discoverer* 3.1	1.93 [1.80–2.06]	< 0.001
	Identificands / *MZmine* 4.5.0	0.13 [0.12–0.15]	< 0.001
1	*Identificands* / *Compound Discoverer* 3.1	0.29 [0.26–0.33]	< 0.001
	MZmine 4.5.0 / *Compound Discoverer* 3.1	1.82 [1.71–1.95]	< 0.001
	Identificands / *MZmine* 4.5.0	0.16 [0.14–0.18]	< 0.001
10	*Identificands* / *Compound Discoverer* 3.1	0.45 [0.41–0.49]	< 0.001
	MZmine 4.5.0 / *Compound Discoverer* 3.1	1.78 [1.68–1.88]	< 0.001
	Identificands / *MZmine* 4.5.0	0.25 [0.23–0.28]	< 0.001

[a] 1 ng/mL corresponds to 0.01 mg/kg in the matrix.

Table S11: Comparison of expected and observed identification outcomes for blind assay analytes based on performance assessment results.

Compound	Blind Assay Outcomes							*Identificands* NTS Identification Outcomes[a,b]											
	Quantitative Results[c] (mg/kg)					*Identificands* NTS Outcomes (Yes/No)		0.005 mg/kg				0.01 mg/kg				0.1 mg/kg			
	Sample 1 (Pinapple)	Sample 2 (Pinapple)	Sample 3 (Chia Seeds)	Sample 4 (Papaya)	Sample 5 (Tomato)	Identified	Expected[d]	Avocado	Lettuce	Orange	Papaya	Avocado	Lettuce	Orange	Papaya	Avocado	Lettuce	Orange	Papaya
Acephate					0.038	No	No	–	–	–	–	–	–	–	–	–	–	–	–
Azoxystrobin				< 0.010		No	No	Yes	–	Yes	–	Yes	Yes	Yes	Yes	Yes	Yes	Yes	Yes
Bifenthrin					< 0.010	No													
Boscalid					< 0.010	No	No	–	–	–	–	–	–	–	–	Yes	Yes	–	Yes
Chlorantraniliprole			< 0.010			No	No	–	–	–	–	–	–	–	–	–	–	–	–
Clothianidin					0.012	No	No	–	–	–	–	–	–	–	–	Yes	Yes	Yes	Yes
Difenoconazole				0.015		No	No	–	–	–	–	–	–	–	–	Yes	–	Yes	Yes
Dinotefuran					< 0.010	No	No	–	–	–	–	–	–	–	–	–	–	–	–
Fipronil					< 0.010	No													
Fluopicolide					< 0.010	No	No	Yes	–	–	–	Yes	–	–	–	Yes	Yes	Yes	Yes
Imidacloprid				0.050		Yes	Yes	–	–	–	–	–	–	–	–	Yes	Yes	Yes	Yes
Imidaclorpid-olefin				0.015		No	No	–	–	–	–	–	–	–	–	Yes	Yes	Yes	Yes
Lambda-Cyhalothrin					< 0.010	No													
Methamidophos					0.020	No	No	–	–	–	–	–	–	–	–	–	–	–	–
Prochloraz				0.398		Yes	Yes	–	–	Yes	–	Yes	Yes	Yes	–	–	–	Yes	–
Propiconazole				0.014		No	No	–	–	–	–	–	–	–	–	Yes	Yes	Yes	Yes
Sulfoxaflor					< 0.010	No	No	–	–	–	–	–	–	–	–	Yes	Yes	Yes	Yes
Tebuconazole				0.024		Yes	Yes	–	–	–	Yes	–	–	–	Yes	Yes	Yes	Yes	Yes
Thiabendazole	< 0.010	0.021				Yes	Yes[e]	–	–	–	–	Yes	Yes	–	Yes	Yes	Yes	Yes	Yes
Thiamethoxam					0.016	No	Yes[f]	–	Yes	–	–	Yes	Yes	–	–	Yes	Yes	Yes	Yes
Trifloxystrobin				0.010		No	Yes	–	–	–	Yes	–	Yes	Yes	Yes	Yes	Yes	Yes	Yes

[a] Binary NTS identification outcomes from the identification performance assessment for analytes found to be common to the blind assay. Yes: positive identification; –: no identification
[b] 1 ng/mL corresponds to 0.01 mg/kg in the matrix.
[c] Quantitative results were obtained using an in-house method validated under SANTE guidelines.
[d] Expected blind assay outcome based on performance assessment results.
[e] Pineapple was not included in the performance evaluation; however, papaya—an extrapolable high-water-content matrix—was included.
[f] Tomato was not assayed in the performance evaluation.

Table S12: NTS identification confidence and identificand probabilities from *Identificands* for selected candidate analytes in avocado extract (10 ng/mL). The candidate groups shown are examples where methodological amenability and the ionized species (protonated species) are relevant.

Compound	Molecular Formula	Exacta Mass	Identification confidence ($p_{\text{identif.}}$)	Identificand Probabilities					
				$p_{\mathcal{I}_{\text{XQ}}}$	$p_{\mathcal{I}_{\text{IP}}}$	$p_{\mathcal{I}_{\text{A}}}$	$p_{\mathcal{I}_{\text{RT}}}$	$p_{\mathcal{I}_{\text{IS}}}$	$p_{\mathcal{I}_{\text{MS2}}}$
Oxasulfuron[a]	$C_{17}H_{18}N_4O_6S$	406.09471	0.752	0.830	0.900	0.924	0.445	0.970	0.880
Ethyl 2-[3-[(4-chloro-2-nitrobenzoyl)amino]-4-methylphenoxy]propanoate	$C_{19}H_{19}ClN_2O_6$	406.09316	0.517	0.830	0.330	0.596	0.161	0.890	0.780
1-Thio-β-D-glucose pentaacetate	$C_{16}H_{22}O_{10}S$	406.09337	0.455	0.830	0.669	0.416	0.438	0.520	0.130
Diclocymet[a]	$C_{15}H_{18}Cl_2N_2O$	312.07962	0.625	0.793	0.376	0.932	0.435	0.940	0.715
6-Amino-2-[(thiophen-2-ylmethyl)amino]-1	$C_{14}H_{12}N_6OS$	312.07933	0.525	0.793	0.961	0.532	0.363	0.940	0.175
4-[(2-Chlorophenyl)azo]-2,4-dihydro-5-methyl-2-phenyl-3H-pyrazol-3-one	$C_{16}H_{13}ClN_4O$	312.07779	0.399	0.793	0.113	0.372	0.446	0.890	0.211
N-[(benzyloxy)carbonyl]-L-Cysteinylglycine	$C_{13}H_{16}N_2O_5S$	312.07799	0.343	0.793	0.134	0.484	0.269	0.443	0.172
2,4-Dihydro-2-(5-((2-hydroxyethyl)sulphonyl)-2-methoxyphenyl)-5-methyl-3H-pyrazol-3-one	$C_{13}H_{16}N_2O_5S$	312.07799	0.341	0.793	0.113	0.580	0.024	0.870	0.204
Tebutam[a]	$C_{15}H_{23}NO$	233.17796	0.750	0.890	0.983	0.872	0.442	1.000	0.816
1-(3,4-dimethylphenyl)-2-(ethylamino)pentan-1-one	$C_{15}H_{23}NO$	233.17796	0.674	0.890	0.983	0.552	0.441	1.000	0.667
3,4-Dihydro-2,2,4,7-tetramethyl-2H-quinoline-1-ethanol	$C_{15}H_{23}NO$	233.17796	0.641	0.890	0.983	0.416	0.445	0.950	0.667
4-(4-Phenylbutyl)piperidin-4-ol	$C_{15}H_{23}NO$	233.17796	0.638	0.890	0.983	0.396	0.417	0.990	0.736
4-(Dibutylamino)benzaldehyde	$C_{15}H_{23}NO$	233.17796	0.621	0.890	0.983	0.432	0.429	0.990	0.552
Meptazinol	$C_{15}H_{23}NO$	233.17796	0.574	0.890	0.983	0.372	0.412	0.990	0.420
1-(Bicyclo[2.2.1]hept-5-en-2-yl)-3-piperidinopropan-1-one	$C_{15}H_{23}NO$	233.17796	0.561	0.890	0.983	0.372	0.427	0.960	0.351
Deoxynupharidine	$C_{15}H_{23}NO$	233.17796	0.521	0.890	0.983	0.396	0.438	0.970	0.175
7-Epi-Deoxynupharidine	$C_{15}H_{23}NO$	233.17796	0.521	0.890	0.983	0.396	0.438	0.970	0.175
Ethoprophos[a]	$C_8H_{19}O_2PS_2$	242.05641	0.683	0.850	0.942	0.844	0.446	1.000	0.505
7-(2-Chloroethyl)theophylline	$C_9H_{11}ClN_4O_2$	242.05705	0.499	0.850	0.241	0.588	0.256	0.930	0.580
Tipiracil	$C_9H_{11}ClN_4O_2$	242.05705	0.474	0.850	0.241	0.532	0.148	0.880	0.677
O,O-dibutyl hydrogen dithiophosphate	$C_8H_{19}O_2PS_2$	242.05641	0.456	0.850	0.942	0.144	0.381	0.510	0.370
O,O-bis(sec-butyl) hydrogen dithiophosphate	$C_8H_{19}O_2PS_2$	242.05641	0.454	0.850	0.942	0.228	0.445	0.480	0.231
Diisobutyl phosphorodithioate	$C_8H_{19}O_2PS_2$	242.05641	0.453	0.850	0.942	0.116	0.446	0.470	0.369
Imidacloprid-dihydroxy-guanidin	$C_9H_{11}ClN_4O_2$	242.05705	0.374	0.850	0.226	0.540	0.000	0.930	0.283
2-Deoxy-D-Glucitol 6-(e)-Vinylhomophosphonate	$C_7H_{15}O_7P$	242.05554	0.218	0.850	0.241	0.052	0.000	0.490	0.045
Edifenphos[a]	$C_{14}H_{15}O_2PS_2$	310.02511	0.734	0.870	0.909	0.780	0.431	0.980	0.841
Dibenzyl hydrogen dithiophosphate	$C_{14}H_{15}O_2PS_2$	310.02511	0.611	0.870	0.909	0.476	0.423	0.900	0.486
O,O-bis(methylphenyl) hydrogen dithiophosphate	$C_{14}H_{15}O_2PS_2$	310.02511	0.544	0.870	0.909	0.524	0.384	0.920	0.217
1,3-Bis(1,1,2,2-tetrafluoroethoxy)benzene	$C_{10}H_6F_8O_2$	310.02400	0.476	0.870	0.567	0.292	0.390	0.736	0.295
3-[(2,4-dihydroxyphenyl)azo]-4-hydroxy-benzenesulfonic acid	$C_{12}H_{10}N_2O_6S$	310.02596	0.434	0.870	0.361	0.296	0.090	0.730	0.686

[a] Target analyte

[b] Data for this table on Zenodo (DOI: 10.5281/zenodo.19135094, file: `Table_S12_Avocado_Identificands.tsv`)

S3 Glossary of Acronyms and Key Technical Terms

S3.1 Machine Learning & Data Science

Area Under Curve (AUC):

A metric used to evaluate machine learning classifiers, representing the area under a performance curve (e.g., precision versus recall, or false positive rate versus recall) across all classification thresholds. It ranges from 0 to 1, where a higher score indicates greater discriminatory power.[7]

Cross-Validation:

A model validation technique used to evaluate machine learning performance and mitigate overfitting. The dataset is partitioned into k subsets (folds); the algorithm is trained on the union of $k-1$ folds and evaluated on the single remaining fold. This process is repeated k times to calculate average model performance.[7,8]

Decision Tree:

A tree-structured predictive model that recursively partitions data using conditional rules. It consists of two fundamental elements: internal nodes, which represent the splitting criteria applied to input features, and leaves, which represent the final outcomes.[8]

Extreme Gradient Boosting (XGBoost):

A scalable, distributed machine learning library implementing the gradient boosting technique. It utilizes parallel tree boosting to achieve high performance in regression, classification, and ranking tasks.[8]

Gradient Boosting:

An ensemble machine learning technique that transforms weak learners (typically decision trees) into a single strong predictive model. Trees are added sequentially, with each new tree trained to correct the residual errors of the existing ensemble.[8]

Histogram of Oriented Gradients (HOG):

A feature descriptor used in computer vision to characterize object shape. It divides an image into spatial cells, computes a 1D histogram of local gradient directions for each, and contrast-normalizes these histograms across larger spatial blocks to form the final representation.[9]

Hyperparameters:

Tunable algorithm configurations that govern the learning process. Unlike model parameters, they cannot be estimated directly from training data and must be optimized to maximize performance.[8]

Input Features:

Properties describing individual entities, used as independent variables to train machine learning models and generate predictions.[7]

Matthews Correlation Coefficient (MCC):

A classification performance metric that provides an unbiased evaluation of imbalanced datasets. It measures the correlation between actual and predicted classifications on a $[-1, 1]$ scale, where 1 indicates perfect classification, 0 represents random guessing, and -1 indicates perfect inverse prediction.[10]

Multiclass Classification Performance (MCP):

A metric used to evaluate multiclass classification tasks, calculated from the predicted probabilities across all k classes.[13]

Multiclass Classification:

A machine learning categorization task that assigns an entity to one of three or more mutually exclusive classes,[8] applied in this study to evaluate peak quality.

Multilabel Classification:

A machine learning categorization task that assigns an entity to multiple non-mutually exclusive classes simultaneously,,[8] applied in this study to infer the ionized species (protonated and deprotonated) formed by a molecule.

Object-Oriented Programming (OOP):

A programming paradigm that organizes software into modular classes. These classes define the properties and behaviors of objects (e.g., chemical compounds or analytes). During execution, objects interact, manipulating data representing their states. This modularity facilitates building, maintaining, modifying, and reusing software.[14]

Random Forest (RF):

An ensemble machine learning algorithm comprising multiple decision trees. For classification, it outputs the majority class; for regression, it outputs the mean prediction.[8]

Recall:

The true positive rate; the proportion of actual positive instances correctly identified by a model..

Wavelet Transform (WT):

A mathematical signal processing tool that enables the analysis of local signal properties across multiple scales, revealing fine details useful for pattern recognition.[11] In this study, WT is applied to filter noise and locate probable regions containing isotopic patterns.[12]

S3.2 Cheminformatics Tools

InChI:

A non-proprietary canonical identifier of chemical substances that can be used in printed and electronic data sources to enable easier linking of diverse data compilations.[15]

InChIKey:

A fixed length (character) condensed digital representation of the InChI that is not human-understandable.[15]

MetFrag:

An open-source computational tool for the *in silico* fragmentation of small molecules, used to annotate candidate compounds and facilitate their identification using tandem mass spectrometry (MS/MS) data.[16]

RDKit & Mordred:

Open-source cheminformatics software libraries used in tandem to parse chemical structures and compute molecular descriptors for machine learning inference.[17,18]

Simplified Molecular Input Line Entry System (SMILES):

A chemical notation language that uses a character sequence to represent a molecular structure as a valence-oriented graph, encoding 2D topology and, optionally, stereochemical configuration.[19,20]

S3.3 Metrological & Analytical Concepts

Chemical Space Reference Library (CRL):

A collection of known and predicted compounds[23] relevant to a specific field. In this study, it comprises potential contaminants identifiable in the analytical sample. The

CRL is implemented as a SQLite database containing the following fields: compound name, canonical SMILES, InChIKey, molecular formula, and neutral monoisotopic mass. The library is fully customizable, allowing users to add or remove compounds based on their specific requirements.

Consensus Reference Set (CRS):

A baseline set of true-positive identifications used to evaluate algorithmic sensitivity independently of matrix effects.

Coverage Factor (k):

Number larger than one by which a combined standard measurement uncertainty is multiplied to obtain an expanded measurement uncertainty.[22]

Examinand:

Nominal property intended to be examined.[21]

Identificand:

A measurand or examinand determined via an analytical method for the purpose of chemical identification.

Identification properties:

The attributes required to establish the chemical identity of a compound, encompassing both qualitative characteristics and quantitative physicochemical measurements.[24]

Measurand:

Quantity intended to be measured.[22]

Methodological Amenability:

The probability of successfully analyzing a compound using a specific analytical method. In this study, it is the probability of a compound being classified as amenable rather

than unamenable, based on its combined suitability for QuEChERS extraction, liquid chromatographic separation, and electrospray ionization.

Nominal property:

Property of a phenomenon, body, or substance, where the property has no size.[21]

Reference Compound:

A candidate chemical structure used to establish the identity of an analyte. Its identification properties are derived experimentally from a reference material or theoretically through database searches and machine learning inference.

Uncertainty Budget:

Statement of a measurement uncertainty, of the components of that measurement uncertainty, and of their calculation and combination.[22]

S3.4 Acronyms & Abbreviations

ACN: Acetonitrile.

ANN: Artificial Neural Network.

AUC-PRC: Area Under the Precision-Recall Curve.

AUC-MCP: Area Under the Multiclass Classification Curve.

CRL: Chemical Space Reference Library.

CRS: Consensus Reference Set.

DDA: Data-Dependent Acquisition.

DIA: Data-Independent Acquisition.

ESI: Electrospray Ionization.

FN: False Negative.

FP: False Positive.

FS: Full Scan.

HESI: Heated Electrospray Ionization.

HOG: Histogram of Oriented Gradients.

HPLC: High-Performance Liquid Chromatography.

HRMS: High-Resolution Mass Spectrometry.

MAE: Mean Absolute Error.

MCC: Matthews Correlation Coefficient.

ML: Machine Learning.

NTS: Non-Targeted Screening.

PCA: Principal Component Analysis.

PR: Probable Regions.

PRM: Parallel Reaction Monitoring.

QuEChERS: Quick, Easy, Cheap, Effective, Rugged, and Safe.

RC: Reference Compound.

RF: Random Forest.

RM: Reference Material.

RMSE: Root-Mean-Square Error.

RT: Retention Time.

SD: Standard Deviation.

SS: Suspect Screening.

TA: Targeted Analysis.

TN: True Negative.

TP: True Positive.

TPP: Triphenylphosphate.

UHPLC: Ultra-High-Performance Liquid Chromatography.

vDIA: Variable Data-Independent Acquisition.

WT: Wavelet Transform.

XGBoost: Extreme Gradient Boosting.

XIC: Extracted Ion Chromatogram.

References

(1) R Core Team, R: A Language and Environment for Statistical Computing. https://www.R-project.org/ (accessed 2026-02-05).

(2) Lenth, R.; Piaskowski, J. emmeans: Estimated Marginal Means, aka Least-Squares Means. https://rvlenth.github.io/emmeans/ (accessed 2026-02-05).

(3) Hartig, DHARMa: Residual Diagnostics for Hierarchical (Multi-Level/Mixed) Regression Models. https://github.com/florianhartig/dharma (accessed 2026-02-05).

(4) Sellers, K. F.; Borle, S.; Shmueli, G. The COM-Poisson model for count data: a survey of methods and applications. *Applied Stochastic Models in Business and Industry* **2012**, *28*, 104–116, DOI: 10.1002/asmb.918.

(5) Fiehn Lab, MoNA - MassBank of North America. https://mona.fiehnlab.ucdavis.edu/ (accessed 2025-12-05).

(6) Chambers, M. C. et al. A cross-platform toolkit for mass spectrometry and proteomics. *Nature Biotechnology* **2012**, *30*, 918–920, DOI: 10.1038/nbt.2377.

(7) Sammut, C.; Webb, G. I. *Encyclopedia of Machine Learning and Data Mining*, 2nd ed.; Springer Science & Business Media: New York, U.S.A., 2017.

(8) Wang, J. *Encyclopedia of Data Science and Machine Learning*; IGI Global Scientific Publishing: Hershey, PA, U.S.A., 2023.

(9) Dalal, N.; Triggs, B. Histograms of Oriented Gradients for Human Detection. 2005 IEEE Computer Society Conference on Computer Vision and Pattern Recognition (CVPR'05). San Diego, CA, USA, 2005; pp 886–893, DOI: 10.1109/CVPR.2005.177.

(10) Grandini, M.; Bagli, E.; Visani, G. Metrics for Multi-Class Classification: an Overview. https://arxiv.org/abs/2008.05756; Version Number: 1.

(11) Poularikas, A. D. *The Transforms and Applications Handbook*, 2nd ed.; CRC Press: Boca Raton, FL, U.S.A., 2000.

(12) Du, P.; Kibbe, W. A.; Lin, S. M. Improved peak detection in mass spectrum by incorporating continuous wavelet transform-based pattern matching. *Bioinformatics (Oxford, England)* **2006**, *22*, 2059–2065, DOI: 10.1093/bioinformatics/btl355.

(13) Aguilar-Ruiz, J. S.; Michalak, M. Multiclass Classification Performance Curve. *IEEE Access* **2022**, *10*, 68915–68921, DOI: 10.1109/ACCESS.2022.3186444.

(14) Weisfeld, M. A. *The object-oriented thought process*, fifth edition ed.; Developer's library; Addison-Wesley: New York, U.S.A., 2019.

(15) Gold, V., McNaught, A., The International Union of Pure and Applied Chemistry (IUPAC),, Eds. *The IUPAC Compendium of Chemical Terminology: The Gold Book*, 5th ed.; International Union of Pure and Applied Chemistry (IUPAC): Research Triangle Park, NC, 2025; DOI: 10.1351/goldbook.

(16) Ruttkies, C.; Neumann, S.; Posch, S. Improving MetFrag with statistical learning of fragment annotations. *BMC Bioinformatics* **2019**, *20*, 376, DOI: 10.1186/s12859-019-2954-7.

(17) Moriwaki, H.; Tian, Y.-S.; Kawashita, N.; Takagi, T. Mordred: A molecular descriptor calculator. *Journal of Cheminformatics* **2018**, *10*, 4, DOI: 10.1186/s13321-018-0258-y.

(18) RDKit, RDKit: Open-source cheminformatics. https://www.rdkit.org (accessed 2025-06-06).

(19) Weininger, D. SMILES, a chemical language and information system. 1. Introduction to methodology and encoding rules. *Journal of Chemical Information and Computer Sciences* **1988**, *28*, 31–36, DOI: 10.1021/ci00057a005.

(20) James, C. A. OpenSMILES specification. http://opensmiles.org/opensmiles.html (accessed 2026-06-20).

(21) Nordin, G.; Dybkaer, R.; Forsum, U.; Fuentes-Arderiu, X.; Pontet, F. Vocabulary on nominal property, examination, and related concepts for clinical laboratory sciences (IFCC-IUPAC Recommendations 2017). *Pure and Applied Chemistry* **2018**, *90*, 913–935, DOI: 10.1515/pac-2011-0613.

(22) BIPM,; IEC,; IFCC,; ILAC,; ISO,; IUPAC,; IUPAP,; OIML, International Vocabulary of Metrology — Basic and general concepts and associated terms, 3rd edition. https://doi.org/10.59161/JCGM200-2012(2012); tex.howpublished: Joint Committee for Guides in Metrology, JCGM 200:2012. (3rd edition).

(23) Milman, B. L.; Zhurkovich, I. K. The chemical space for non-target analysis. *TrAC Trends in Analytical Chemistry* **2017**, *97*, 179–187, DOI: 10.1016/j.trac.2017.09.013.

(24) Milman, B. L. *Chemical identification and its quality assurance*, 1st ed.; Springer: Berlin Heidelberg, 2011; DOI: 10.1007/978-3-642-15361-7.
